# Supplementary material for: Social reward outcompetes drug seeking dopaminergic ensembles to prevent relapse
Source: Nat Commun. 2026 Apr 3;17:3462. doi: 10.1038/s41467-026-71357-4 (PMC13076667; doi:10.1038/s41467-026-71357-4)
Supplement: Supplementary file 1 — Supplementary Information [file 41467_2026_71357_MOESM1_ESM.pdf]

Supplemental information

Social Reward Outcompetes Drug Seeking Dopaminergic Ensembles to Prevent Relapse

Wei Zheng[1,†], Xiaoxing Liu[2,†], Tangsheng Lu[3,†], Xinyou Lv[4], Xuefang Guan[5], Yifan Yu[2], Xue Li[3], Zhe Wang[6], Kai Yuan[2], Jeffrey W. Grimm[7], Trevor W. Robbins[8,9], Jie Shi[3,*], Lin Lu[1,2,3,6,*], Yan-Xue Xue[3,10,*]

*Email: shijie@bjmu.edu.cn; linlu@bjmu.edu.cn; yanxuexue@bjmu.edu.cn

Supplementary Figures

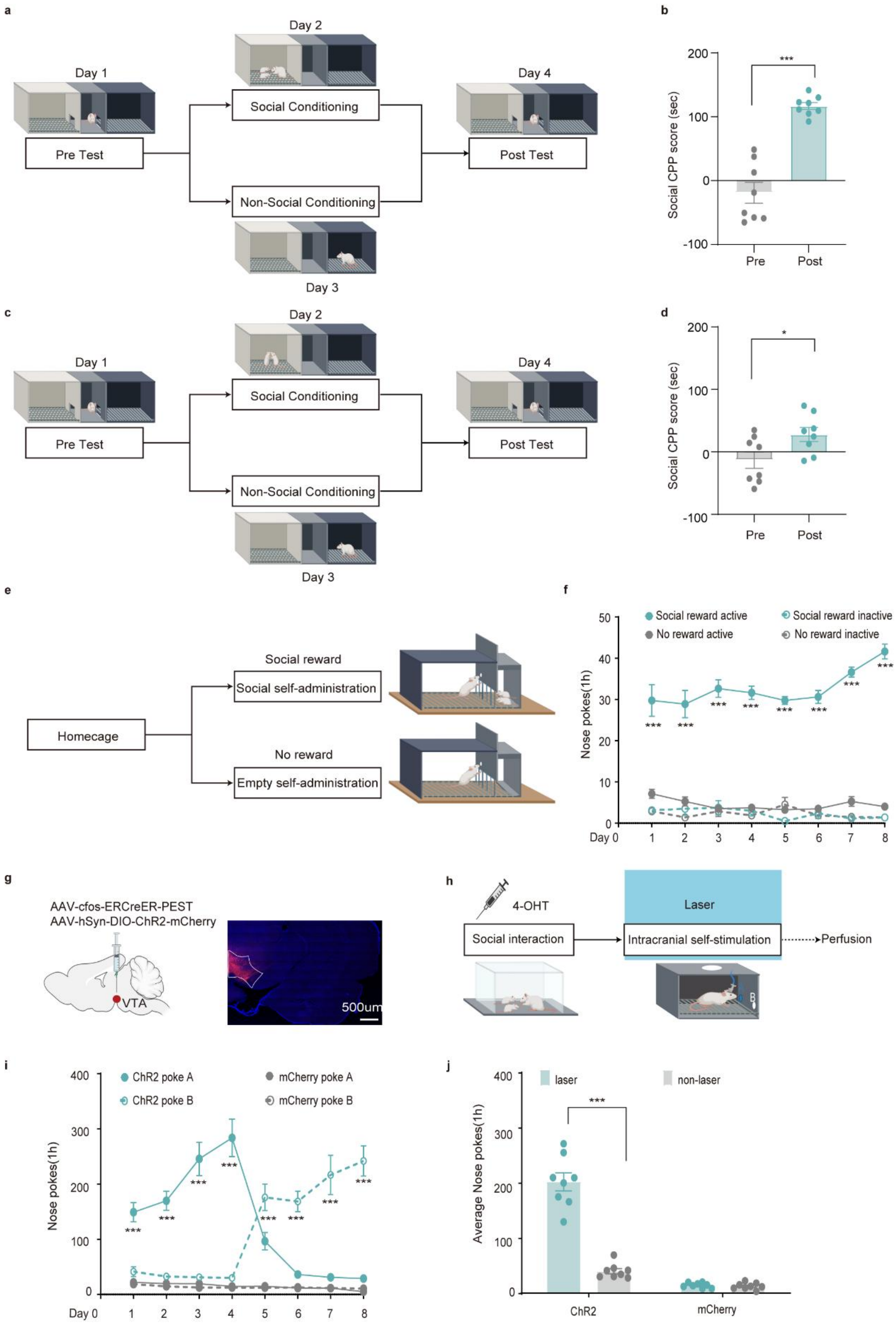

Supplementary Fig.1 | Interacting with two juvenile partners is rewarding. a, Experimental timeline for social conditioned place preference (CPP) induced by interaction with two juvenile partners. **b**, Social CPP scores for baseline preference and the expression test. (*n* = 8 male rats, paired two-tailed t test, $t_{(7)} = 9.554$, $p < 0.001$). **c**, Experimental timeline for social CPP induced by interaction with an age-matched partner. **d**, Social CPP scores for baseline preference and the expression test (*n* = 8 male rats, paired two-tailed t test, $t_{(7)} = 3.200$, $p = 0.015$). **e**, Experimental timeline for social self-administration induced by interaction with two juvenile partners. **f**, Number of nose pokes during social self-administration sessions (*n* = 8 male rats, three-way ANOVA: $F_{(7,8)} = 3.781$, $p = 0.041$; more statistics see Supplementary Table 12A). **g**, Virus injection strategy (repeated 4 times independently with similar results; scale bar, 500 μm). **h**, Experimental timeline for optical intracranial self-stimulation (ICSS). **i**, Active nose pokes at port A and port B during each 60-min ICSS session (*n* = 8 male rats, three-way ANOVA: day1-4, $F_{(3,12)} = 5.764$, $p = 0.011$, more statistics see Supplementary Table 12B; day5-8, $F_{(3,12)} = 21.248$, $p < 0.001$, more statistics see Supplementary Table 12C). **j**, Mean number of nose pokes during laser and non-laser trials, averaged across all training days. Elevated mean laser-evoked nose pokes relative to non-laser trials were observed exclusively in ChR2-expressing rats (*n* = 8 male rats, two-way ANOVA with simple effect analysis: $F_{(1,14)} = 103.654$, $p < 0.001$; more statistics see Supplementary Table 12D). All the data are presented as mean ± SEM. 95% confidence interval was used for all statistical analyses in this figure. Panels a, c,

e, g, h: Created in BioRender. Xiaoxing, L. (2026) https://BioRender.com/hqs0eg6. $^{*}p < 0.05$, $^{**}p < 0.01$, $^{***}p < 0.001$.

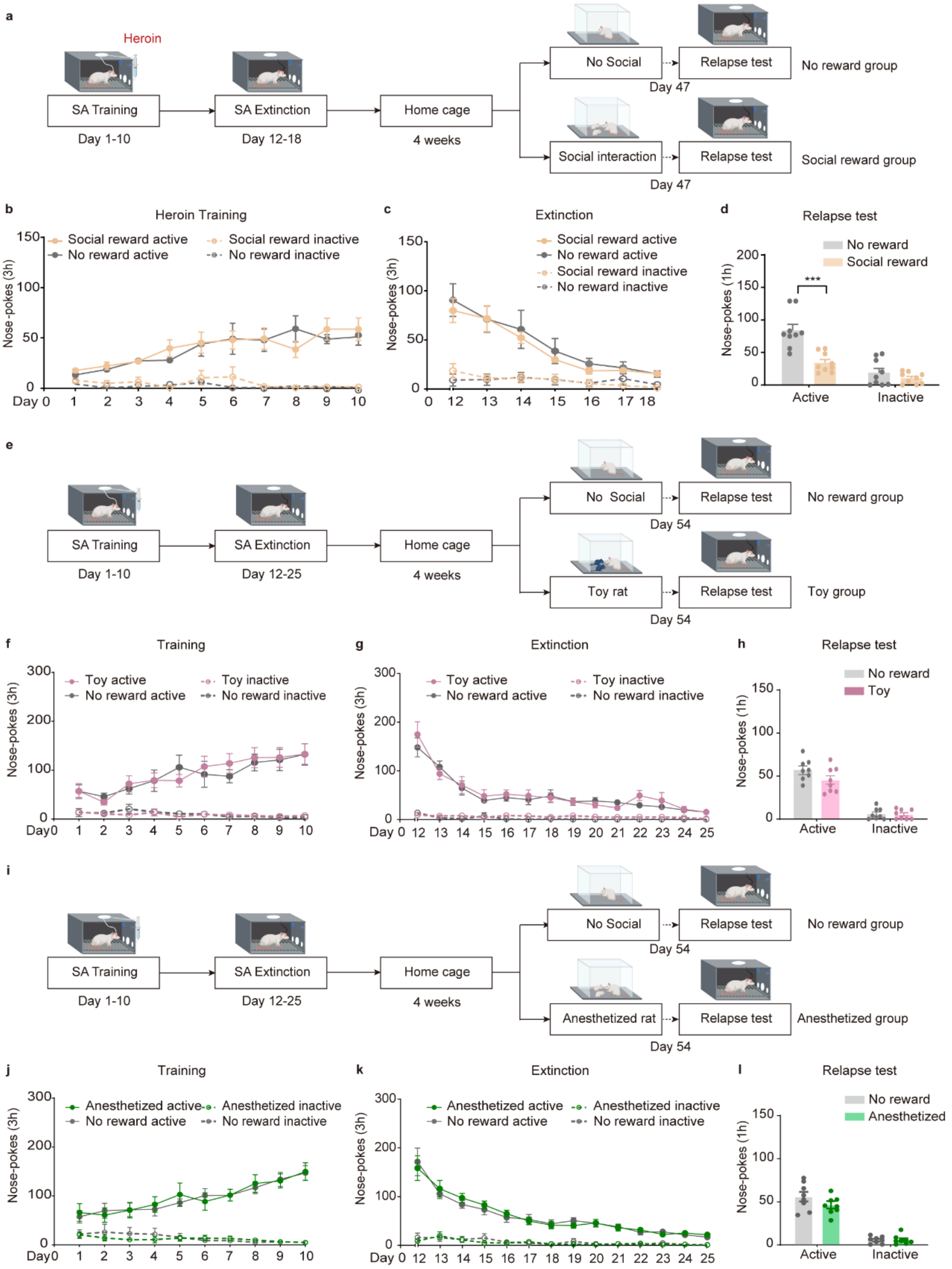

Supplementary Fig.2 | Social reward suppresses drug seeking during the relapse test. a, Schematic representation of the experimental design. **b**, Number of nose-pokes during heroin self-administration training (n = 9 male rats, three-way ANOVA: $F_{(9,8)}$ = 1.582, p = 0.265; more statistics see Supplementary Table 1D). **c**, Number of nose-

pokes during extinction ($n = 9$ male rats, three-way ANOVA: $F_{(6,11)} = 2.739, p = 0.070$; more statistics see Supplementary Table 1E). **d**, Number of nose-pokes during relapse test ($n = 9$ male rats, two-way ANOVA: $F_{(1,16)} = 16.919, p < 0.001$; more statistics see Supplementary Table 1F). **e**, Schematic representation of the experimental design. **f**, Number of nose-pokes during cocaine self-administration training ($n = 8$ male rats, three-way ANOVA: $F_{(9,6)} = 0.397, p = 0.897$; more statistics see Supplementary Table 2A). **g**, Number of nose-pokes during extinction ($n = 8$ male rats, three-way ANOVA: $F_{(13,2)} = 0.962, p = 0.619$; more statistics see Supplementary Table 2B). **h**, Number of nose-pokes during relapse test ($n = 8$ male rats, two-way ANOVA: $F_{(1,14)} = 2.168, p = 0.163$; more statistics see Supplementary Table 2C). **i**, Schematic representation of the experimental design. **j**, Number of nose-pokes during cocaine self-administration training ($n = 8$ male rats, three-way ANOVA: $F_{(9,6)} = 0.284, p = 0.955$; more statistics see Supplementary Table 2D). **k**, Number of nose-pokes during extinction ($n = 8$ male rats, three-way ANOVA: $F_{(13,2)} = 1.011, p = 0.602$; more statistics see Supplementary Table 2E). **l**, Number of nose-pokes during relapse test ($n = 8$ male rats, two-way ANOVA: $F_{(1,14)} = 2.339, p = 0.148$; more statistics see Supplementary Table 2F). All the data are presented as mean ± SEM. 95% confidence interval was used for all statistical analyses in this figure. Panels a, e, i: Created in BioRender. Xiaoxing, L. (2026) https://BioRender.com/hqs0eg6. $^{*}p < 0.05$, $^{**}p < 0.01$, $^{***}p < 0.001$.

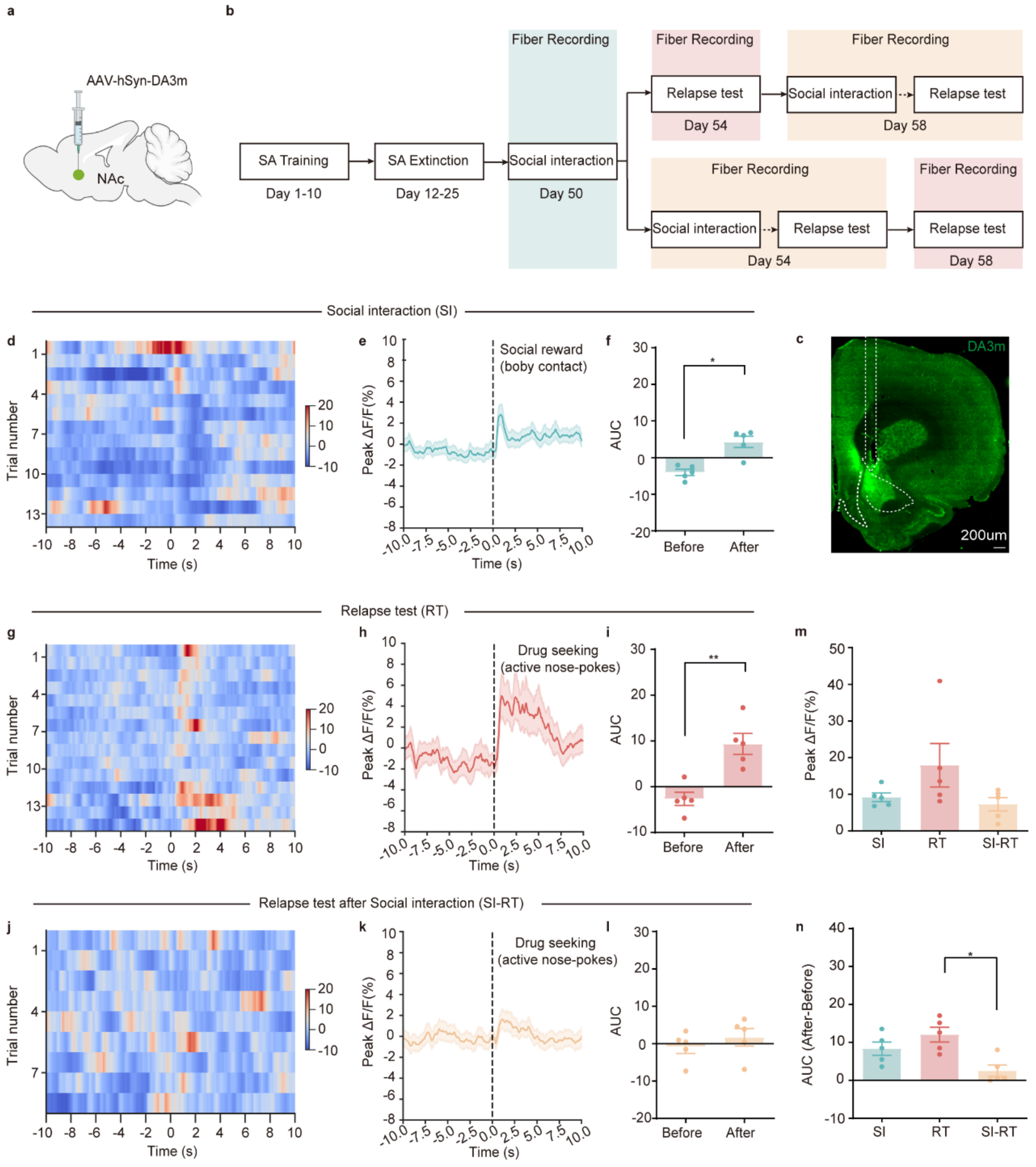

Supplementary Fig.3 | Social reward inhibits DA release in the NAc induced by cocaine seeking. a, Strategy for virus injection (Created in BioRender. Xiaoxing, L. (2026) https://BioRender.com/hqs0eg6). AAV-hSyn-DA3m was injected into the NAc, along with the optical fiber in the same location. **b**, Schematic of the experimental procedure for fiber photometry recording. Fiber recordings were conducted across social interaction (SI), relapse test (RT), and relapse test after social interaction (SI-RT)

sessions in the same animals. To control for potential time effects, animals were divided into two groups: one underwent the cocaine relapse test first, while the other received social interaction prior to the relapse test. Data from both groups were then pooled for analysis. **c**, Representative image of DA3m expression in the VTA (repeated 4 times independently with similar results; scale bar, 200 μm). Green: DA3m. **d**, **g**, **j**, Trial-by-trial heatmap representations of calcium signals during the various behavioral manipulations. Color scales on the right indicate ΔF/F. **e**, **h**, **k**, Event plot of average calcium signals aligned to distinct behavioral manipulation. **f**, **i**, **l**, Comparison of the area under curve (AUC) between baseline (10 s before stimuli) and stimuli (within 10 s from onset of stimuli) of the DA release signals in response to different stimuli ($n = 5$ male rats, paired two-tailed t test: SI, $t_{(4)} = 4.354, p = 0.012$; RT, $t_{(4)} = 6.230, p = 0.003$; SI-RT, $t_{(4)} = 1.653$, $p = 0.174$). **m**, Quantification of peak ΔF/F changes from three sessions: SI, RT, and SI-RT ($n = 5$ male rats, one-way ANOVA with Bonferroni multiple comparisons test, $F_{(2,8)} = 3.555, p = 0.088$). **n**, Quantification of AUC changes from three sessions: SI, RT, and SI-RT ($n = 5$ male rats, one-way ANOVA with Bonferroni multiple comparisons test, $F_{(2,8)} = 13.96, p = 0.003$; RT vs SI-RT, $p = 0.019$). All the data are presented as mean ± SEM. 95% confidence interval was used for all statistical analyses in this figure. $^{*}p < 0.05$, $^{**}p < 0.01$, $^{***}p < 0.001$.

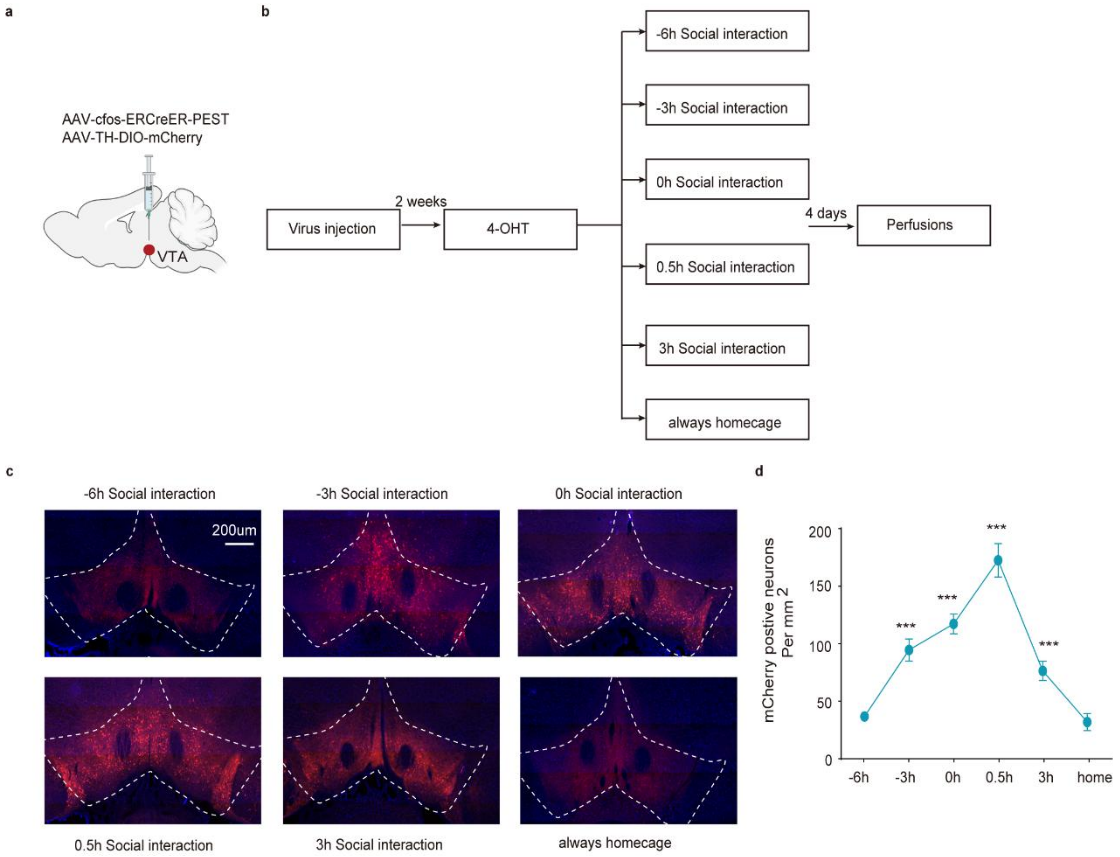

Supplementary Fig.4 | Time course of targeted recombination in active populations (TRAPing). a, Strategy for virus injection (Created in BioRender. Xiaoxing, L. (2026) https://BioRender.com/hqs0eg6). **b**, Timeline of social stimulation experiment to determine effective TRAPing window. **c**, Representative images of TRAPed cells in the ventral tegmental area (VTA) of rats subjected to the social stimulation experiment (repeated 4 times independently with similar results; scale bar, 200 μm). **d**, Quantification of TRAPed cell density in the VTA (n = 4 male rats, one-way ANOVA with bonferroni multiple comparisons test: $F_{(5,18)} = 132.4$, $p < 0.001$; home vs 3h, $p < 0.001$; home vs 0.5h, $p < 0.001$; home vs 0h, $p < 0.001$; home vs -3h, $p < 0.001$; home vs -6h, $p = 0.973$). All the data are presented as mean ± SEM. 95%

confidence interval was used for all statistical analyses in this figure. $^{*}p < 0.05$, $^{**}p < 0.01$, $^{***}p < 0.001$.

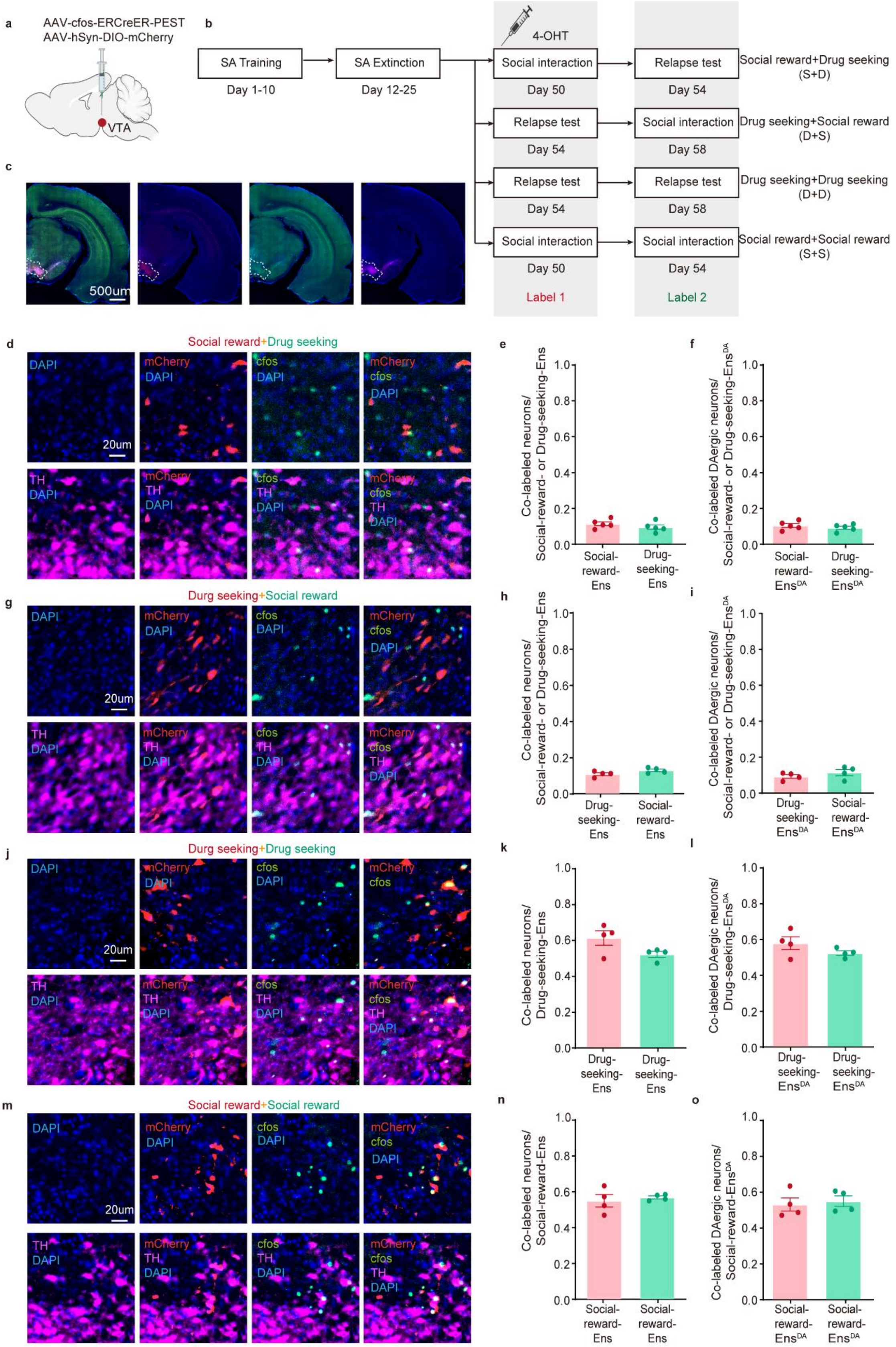

a
AAV-cfos-ERCreER-PEST
AAV-hSyn-DIO-mCherry
VTA
b
SA Training
Day 1-10
SA Extinction
Day 12-25
4-OHT
Social interaction
Day 50
Relapse test
Day 54
Social reward+Drug seeking (S+D)
Relapse test
Day 54
Social interaction
Day 58
Drug seeking+Social reward (D+S)
Relapse test
Day 54
Relapse test
Day 58
Drug seeking+Drug seeking (D+D)
Social interaction
Day 50
Social interaction
Day 54
Social reward+Social reward (S+S)
Label 1
Label 2
c
500um
d
Social reward+Drug seeking
DAPI
mCherry
cfos
TH
20um
e
Co-labeled neurons/ Social-reward- or Drug-seeking-Ens
Social-reward-Ens
Drug-seeking-Ens
f
Co-labeled DAergic neurons/ Social-reward- or Drug-seeking-Ens^DA
Social-reward-Ens^DA
Drug-seeking-Ens^DA
g
Durg seeking+Social reward
h
i
Drug-seeking-Ens
Social-reward-Ens
j
Durg seeking+Drug seeking
k
Co-labeled neurons/ Drug-seeking-Ens
l
Co-labeled DAergic neurons/ Drug-seeking-Ens^DA
m
Social reward+Social reward
n
Co-labeled neurons/ Social-reward-Ens
o
Co-labeled DAergic neurons/ Social-reward-Ens^DA

Supplementary Fig.5 | Social-reward- and Drug-seeking-responsive DAergic ensembles in the VTA are mainly distinct. **a**, **b**, Schematic of the experimental procedure. **c**, Representative sections showing viral expression (red), cfos–positive neurons (green), and tyrosine hydroxylase (TH) positive neurons (pink) (repeated 4 times independently with similar results; scale bar, 500 μm). **d**, Representative images of TH staining in Label 1 and Label 2 (repeated 4 times independently with similar results; scale bar, 20 μm). Red: mCherry-expressed positive neurons (Label 1, Social-reward-responsive ensembles); Green: cfos-expressed positive neurons (Label 2, Drug-seeking-responsive ensembles); Pink: TH-expressed positive neurons; Blue: DAPI. **e**, Percentage of co-labeled neurons in Label 1 or Label 2 (*n* = 5 male rats). **f**, Percentage of co-labeled DAergic neurons in Label 1 or Label 2 (*n* = 5 male rats). **g**, Representative images of TH staining in Label 1 and Label 2 (repeated 4 times independently with similar results; scale bar, 20 μm). Red: mCherry-expressed positive neurons (Label 1, Drug-seeking-responsive ensembles); Green: cfos-expressed positive neurons (Label 2, Social-reward-responsive ensembles). **h**, Percentage of co-labeled neurons in Label 1 or Label 2 (*n* = 4 male rats). **i**, Percentage of co-labeled DAergic neurons in Label 1 or Label 2 (*n* = 4 male rats). **j**, Representative images of TH staining in the Drug-seeking-responsive ensembles (Label 1 versus Label 2, repeated 4 times independently with similar results; scale bar, 20 μm). **k**, Percentage of co-labeled neurons in Label 1 or Label 2 (*n* = 4 male rats). **l**, Percentage of co-labeled DAergic neurons in Label 1 or Label 2 (*n* = 4 male rats). **m**, Representative images of TH staining in the Social-reward-responsive ensembles (Label 1 versus Label 2, repeated 4 times independently with

similar results; scale bar, 20 μm). **n**, Percentage of co-labeled neurons in Label 1 or Label 2 (n = 4 male rats). **o**, Percentage of co-labeled DAergic neurons in Label 1 or Label 2 (n = 4 male rats). All the data are presented as mean ± SEM. 95% confidence interval was used for all statistical analyses in this figure. Panels a, b: Created in BioRender. Xiaoxing, L. (2026) https://BioRender.com/hqs0eg6.

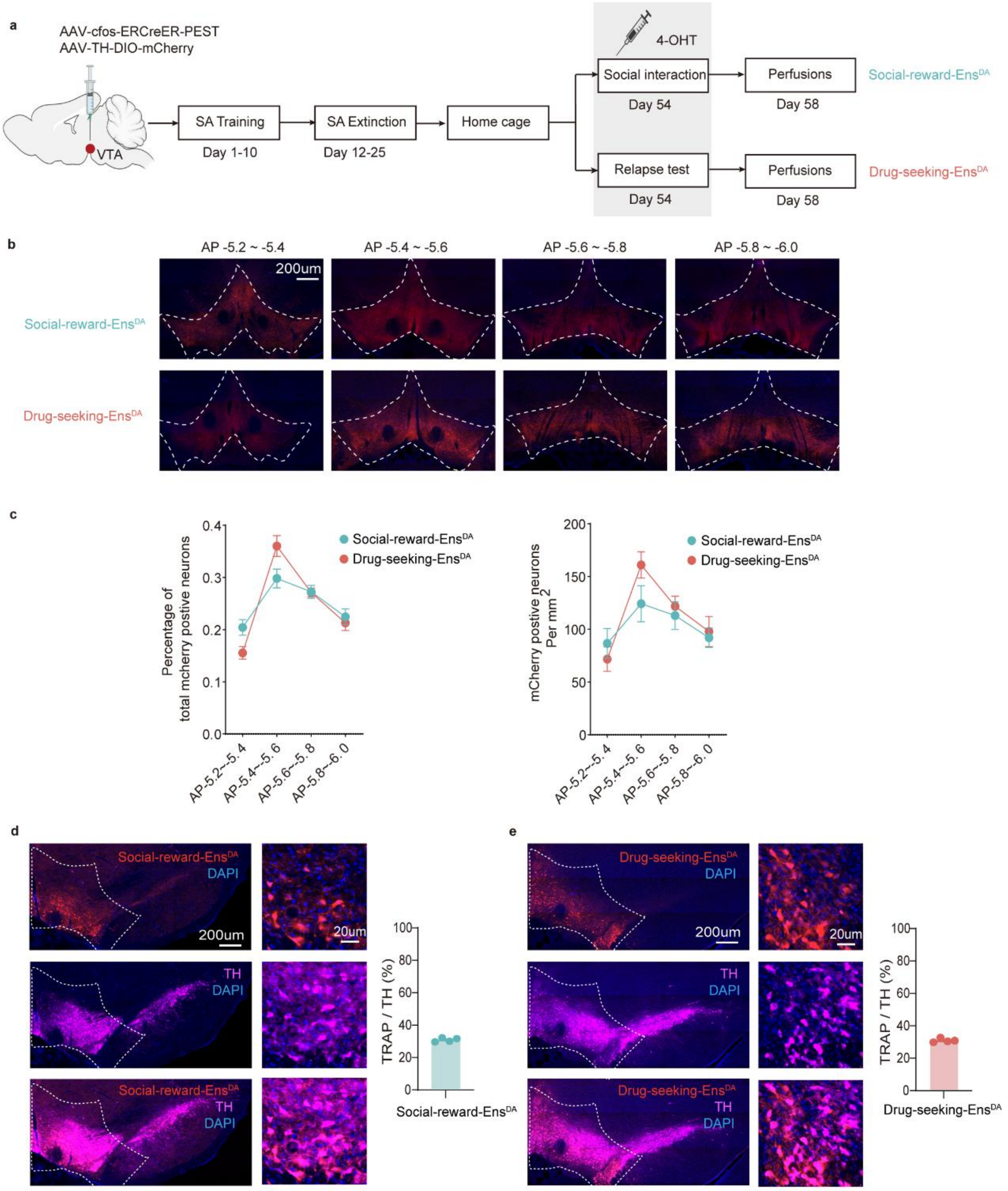

Supplementary Fig.6 | Spatial distributions of VTA DAergic ensembles activated by social reward or drug seeking. a, Schematic of the experimental procedure (Created in BioRender. Xiaoxing, L. (2026) https://BioRender.com/hqs0eg6) used to label VTA DAergic ensembles activated by either social reward or drug seeking. **b**, Representative images showing the distribution of activated DAergic ensembles in the VTA along the anterior–posterior axis (repeated 4 times independently with similar

results; scale bar, 200 μm). Red, c-Fos; blue, DAPI. Dashed lines indicate the boundaries of the VTA. **c**, Quantification of the density of DAergic ensembles (number per mm²) (*n* = 4 male rats, two-way ANOVA Simple effect analysis: $F_{(3,18)} = 5.372$, $p = 0.008$; AP-5.2 ~ -5.4, $p = 0.906$; AP-5.4 ~ -5.6, $p = 0.447$; AP-5.6 ~ -5.8, $p = 0.977$; AP-5.8 ~ -6.0, $p = 0.996$) and relative distribution of DAergic ensembles, expressed as a percentage of the total number of activated DAergic ensembles along the anterior-posterior axis of the VTA (*n* = 4 male rats, two-way ANOVA simple effect analysis: $F_{(3,18)} = 3.625$, $p = 0.033$; AP-5.2 ~ -5.4, $p = 0.170$; AP-5.4 ~ -5.6, $p = 0.222$; AP-5.6 ~ -5.8, $p = 0.999$; AP-5.8 ~ -6.0, $p = 0.974$). **d**, Representative images showing activated DAergic neuronal ensembles in the VTA during social interaction, with quantification of the proportion of these ensembles relative to the total dopamine neuron population in the VTA (repeated 4 times independently with similar results; scale bar, 200 μm; *n* = 4 male rats). **e**, Representative images showing activated DAergic neuronal ensembles in the VTA during drug relapse, with quantification of the proportion of these ensembles relative to the total dopamine neuron population in the VTA (repeated 4 times independently with similar results; scale bar, 200 μm; *n* = 4 male rats). All the data are presented as mean ± SEM. 95% confidence interval was used for all statistical analyses in this figure.

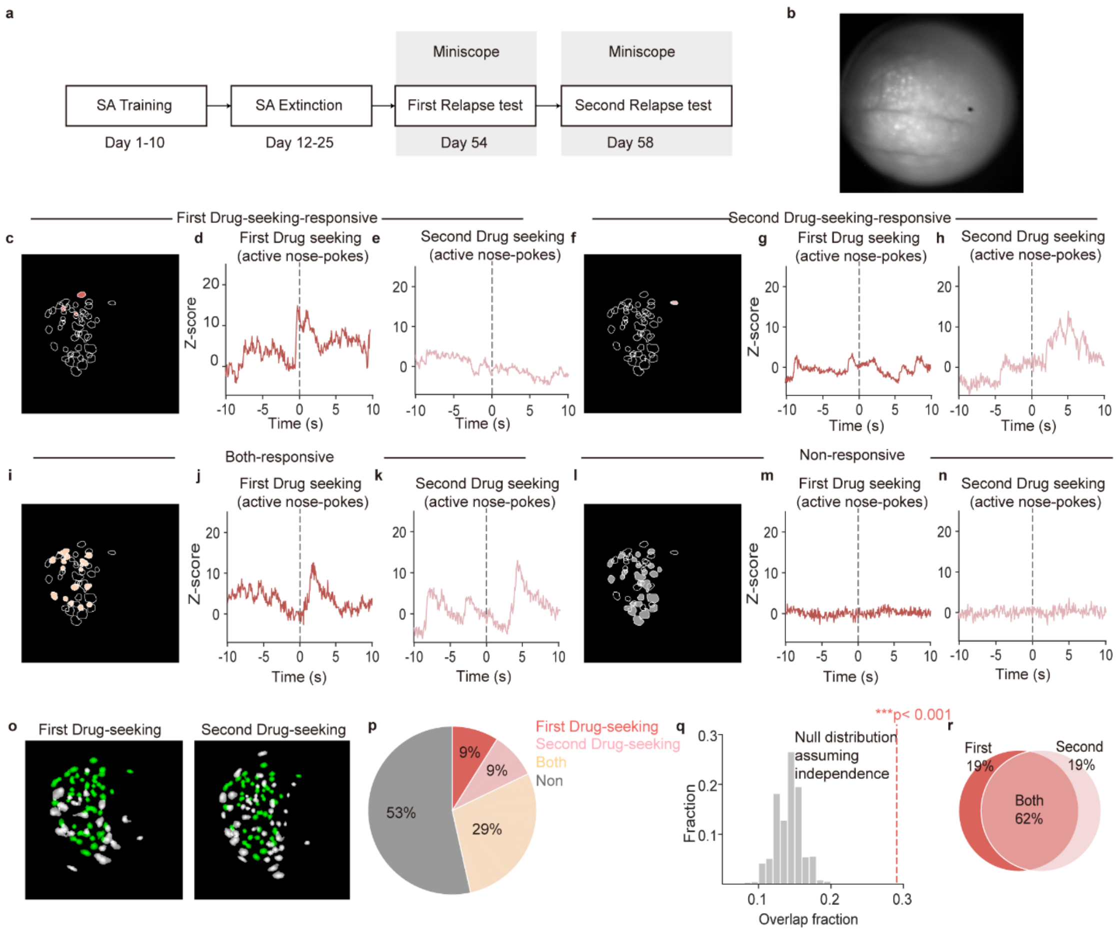

Supplementary Fig.7 | Overlap in VTA DAergic ensemble responses to drug seeking across two relapse tests conducted on separate days. a, Schematic of the experimental design for in vivo calcium imaging. Single-cell calcium imaging was performed in the same animals during the first (Day 54) and the second drug seeking sessions (Day 58). **b**, Representative field of view showing imaged neurons. **c**, **f**, **i**, **l**, Example fields of view with neurons color-coded according to stimulus responsiveness (n = 157 neurons). **d**, **e**, Example of a neuron responsive to the first, but not the second, drug seeking. **g**, **h**, Example of a neuron responsive to the second, but not the first, drug seeking. **j**, **k**, Example of a neuron responsive to both the first and second drug seeking. **m**, **n**, Example of a neuron unresponsive to either drug seeking sessions. **o**, Matched

neurons imaged across both sessions (first and second drug seeking). **p**, Proportions of neurons responsive to first drug seeking, second drug seeking, both, or neither ($n = 157$ neurons). **q**, Observed overlap compared to a null distribution assuming independence between first and second drug-seeking-responsive neuron populations ($n = 157$ neurons, nonparametric permutation test, one-tailed, $p < 0.001$). **r**, Proportion of neurons exhibiting overlapping versus distinct DAergic ensemble activity patterns across the two drug seeking sessions. All the data are presented as mean ± SEM. 95% confidence interval was used for all statistical analyses in this figure. $^{*}p < 0.05$, $^{**}p < 0.01$, $^{***}p < 0.001$.

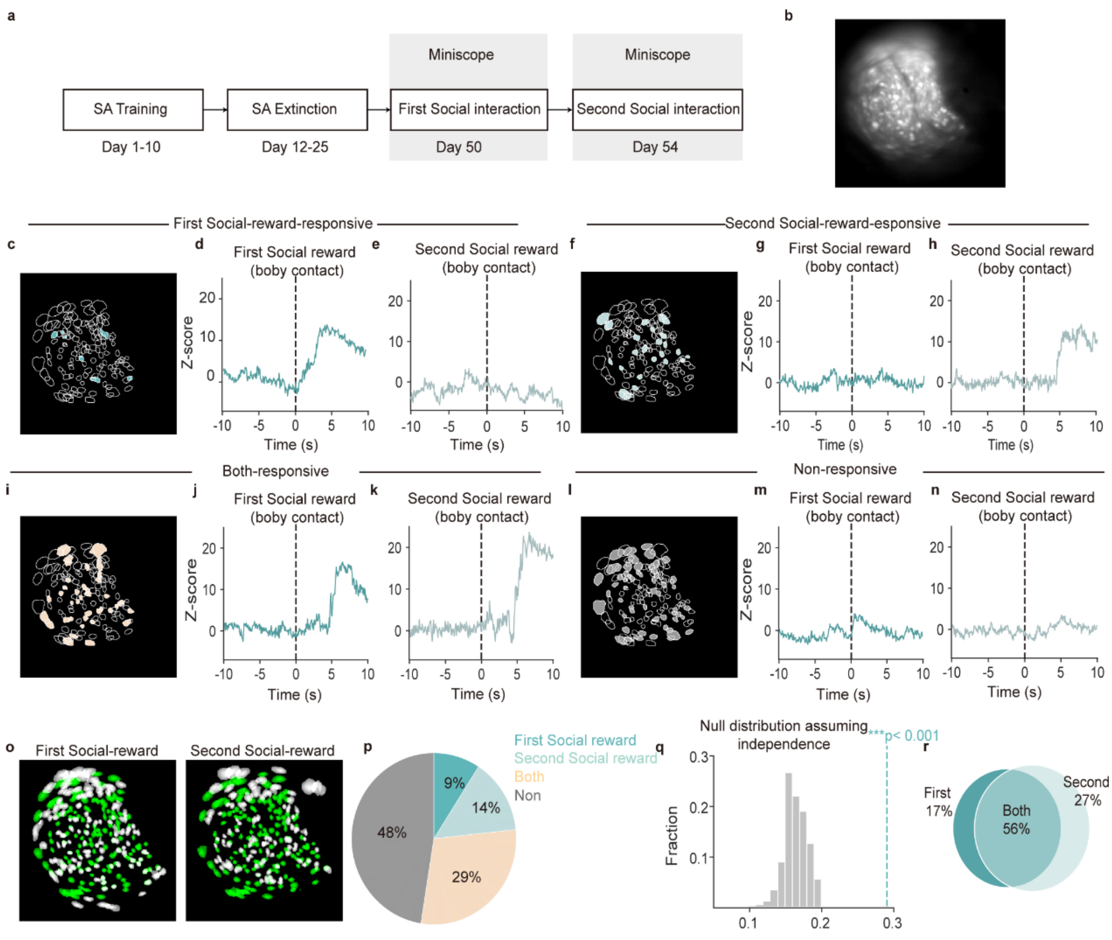

Supplementary Fig.8 | Overlap in VTA DAergic ensemble responses to social reward across two interaction tests conducted on separate days. a, Schematic of the experimental design for in vivo calcium imaging. Single-cell calcium imaging was performed in the same animals during the first (Day 50) and the second social reward sessions (Day 54). **b**, Representative field of view showing imaged neurons. **c**, **f**, **i**, **l**, Example fields of view with neurons color-coded according to stimulus responsiveness ($n = 219$ neurons). **d**, **e**, Example of a neuron responsive to the first, but not the second, social reward. **g**, **h**, Example of a neuron responsive to the second, but not the first, social reward. **j**, **k**, Example of a neuron responsive to both social reward sessions. **m**, **n**, Example of a neuron unresponsive to either session. **o**, Matched neurons imaged

across both sessions (first and second social reward sessions). **p**, Proportions of neurons responsive to first social reward, second social reward, both, or neither (n = 219 neurons). **q**, Observed overlap compared to a null distribution assuming independence between social-reward-responsive neuron populations across sessions (n = 219 neurons, nonparametric permutation test, one-tailed, $p < 0.001$). **r**, Proportion of neurons exhibiting overlapping versus distinct DAergic ensemble activity patterns across the two social reward sessions. All the data are presented as mean ± SEM. 95% confidence interval was used for all statistical analyses in this figure. $^{*}p < 0.05$, $^{**}p < 0.01$, $^{***}p < 0.001$.

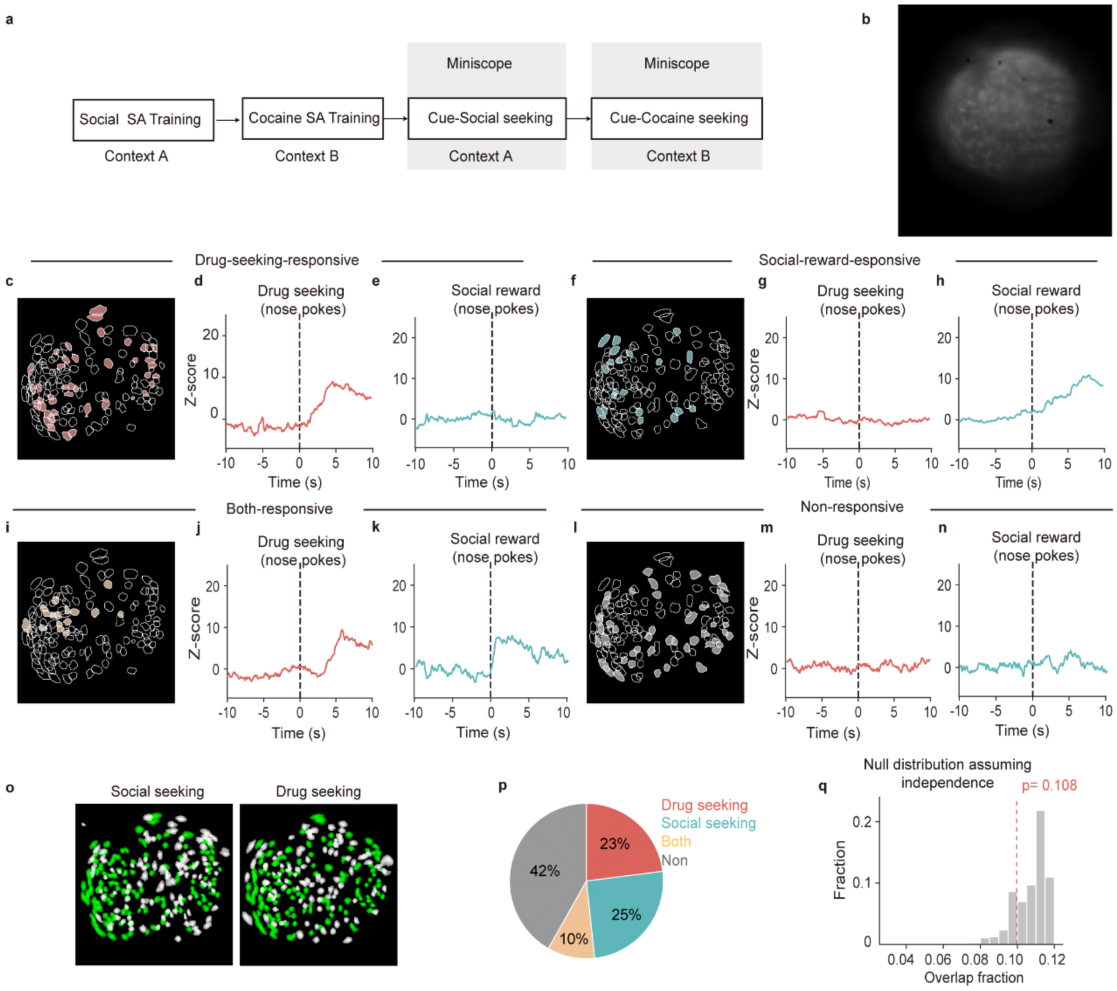

Supplementary Fig.9 | Distinct VTA DAergic ensembles respond to social seeking and drug seeking. a, Schematic of the experimental design for microscope calcium imaging. **b**, Representative raw microscope image. **c**, **f**, **i**, **l**, Example field of view with neurons colored by the stimulus type that they are responsive to. **d**, **e**, Example of a neuron selectivity responsive to cocaine seeking. **g**, **h**, Example of a neuron selectivity responsive to social seeking. **j**, **k**, Example of a neuron responsive to both. **m**, **n**, Example of a neuron responsive to neither. **o**, Matching neurons imaged across distinct sessions (n = 261/519 neurons). **p**, Percentage of neurons responsive to social seeking, cocaine seeking, both, or neither (n = 261 neurons). **q**, Overlap compared with null distribution assuming Social-seeking- and Drug-seeking-responsive neurons are

independent samples ($n = 261$ neurons, nonparametric permutation test, one-tailed, $p = 0.108$). All the data are presented as mean ± SEM. 95% confidence interval was used for all statistical analyses in this figure. $^{*}p < 0.05$, $^{**}p < 0.01$, $^{***}p < 0.001$.

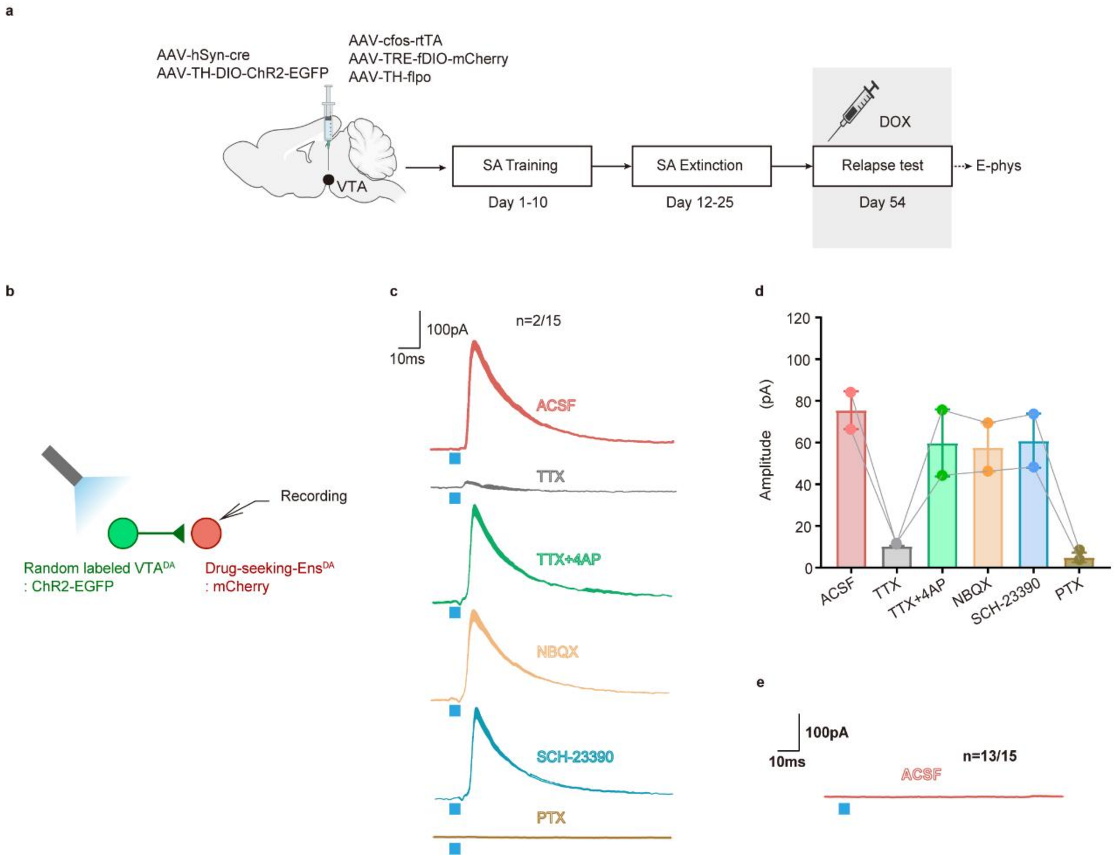

Supplementary Fig.10 | Most Drug-seeking-Ens^{DA} did not exhibit IPSCs upon activation of randomly labeled VTA DAergic neurons. a, Schematic of the experimental procedure (Created in BioRender. Xiaoxing, L. (2026) https://BioRender.com/hqs0eg6) for activation of random-labeled VTA DAergic neurons and electrophysiology recording in Drug-seeking-Ens^{DA}. Rats received Dox injection prior to the relapse test to label Drug-seeking-Ens^{DA} (mCherry). Light-activated ChR2-EGFP in randomly labeled VTA DAergic neurons, and we performed whole-cell voltage-clamp recordings from Drug-seeking-Ens^{DA}. **b**, Cartoon representing patch recording strategy. **c**, Representative traces of IPSCs recorded in ~13% of Drug-seeking-Ens^{DA} following optogenetic stimulation of randomly labeled VTA DAergic neurons. IPSCs were abolished by tetrodotoxin (TTX, gray), restored by

4-aminopyridine (4-AP, green) in the presence of TTX, nd blocked by the $GABA_A$ receptor antagonist picrotoxin (PTX, brown). The currents were unaffected by the AMPA receptor antagonist NBQX (yellow) or the D1receptor antagonist SCH-23390. **d**, IPSC amplitudes under various conditions (ACSF, TTX, TTX+4-AP, NBQX, SCH-23390, or PTX), normalized to baseline (n = 2 neurons; one-way ANOVA with Bonferroni multiple comparisons: $F_{(5,5)} = 21.62$, $p = 0.002$; ACSF vs TTX, $p = 0.005$; TTX vs TTX+4-AP, $p = 0.018$; ACSF vs PTX, $p = 0.004$). **e**, In ~ 87% of Drug-seeking-Ens^{DA} in the VTA, optogenetic stimulation of randomly labeled VTA DAergic neurons did not elicit IPSCs (n = 13 neurons). All the data are presented as mean ± SEM. 95% confidence interval was used for all statistical analyses in this figure. $^{*}p < 0.05$, $^{**}p < 0.01$, $^{***}p < 0.001$.

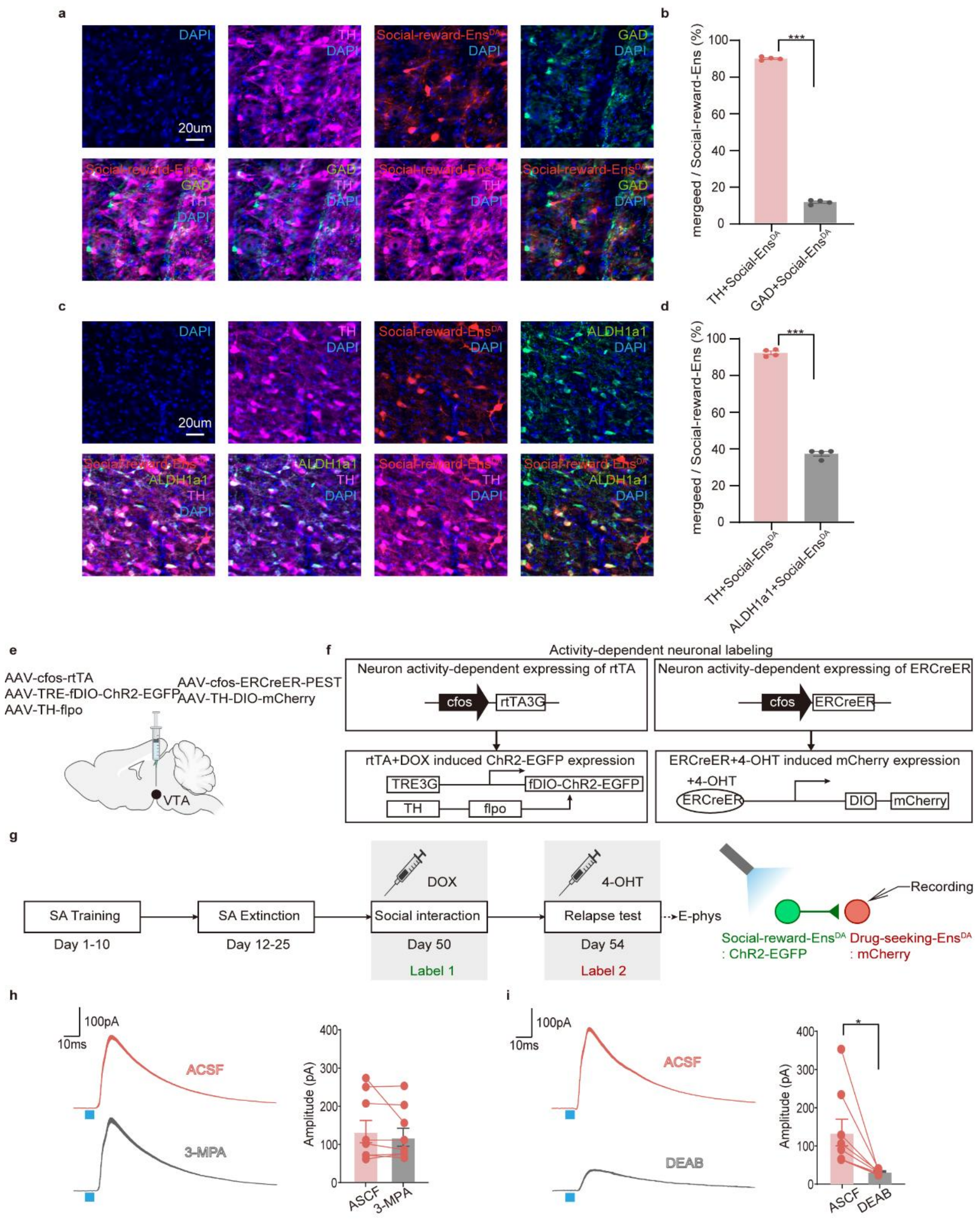

Supplementary Fig.11 | Aldehyde Dehydrogenase 1a1 Mediates the inhibitory effect of Social-reward-Ens^{DA} on Drug-seeking-Ens^{DA}. a, Representative histology images of Social-reward-Ens^{DA} co-label TH or GAD (repeated 4 times independently with similar results; scale bar, 20 μm). **b**, The proportion of co-labeled neurons (TH + Social-reward-Ens^{DA} vs GAD + Social-reward-Ens^{DA}) represents the total Social-

reward-Ens^{DA} (n = 4 male rats, paired two-tailed t test: $t_{(3)} = 155.3$, $p < 0.001$) **c**, Representative histology images of Social-reward-Ens^{DA} co-label TH or ALDH1a1 (repeated 4 times independently with similar results; scale bar, 20 μm). **d**, The proportion of co-labeled neurons (TH + Social-reward-Ens^{DA} vs ALDH1a1 + Social-reward-Ens^{DA}) represents the total Social-reward-Ens^{DA} (n = 4 male rats, paired two-tailed t test: $t_{(3)} = 29.61$, $p < 0.001$). **e**,**f**, Strategy for Virus injection. **g**, Schematic of the experimental procedure for electrophysiology recording. **h**, Representative traces of evoked IPSCs and normalized amplitudes upon bath application of the GAD inhibitor 3-mercaptopropionic acid (3-MPA, 500 μM) compared with control conditions (n = 8 neurons, paired two-tailed t test: $t_{(7)} = 1.049$, $p = 0.329$). **i**, Representative traces of evoked IPSCs and normalized amplitudes upon bath application of the ALDH inhibitor 4-(diethylamino)-benzaldehyde (DEAB, 10 μM) compared with control conditions (n = 8 neurons, paired two-tailed t test: $t_{(7)} = 3.029$, $p = 0.019$). All the data are presented as mean ± SEM. 95% confidence interval was used for all statistical analyses in this figure. Panels e, g: Created in BioRender. Xiaoxing, L. (2026) https://BioRender.com/hqs0eg6. $^{*}p < 0.05$, $^{**}p < 0.01$, $^{***}p < 0.001$.

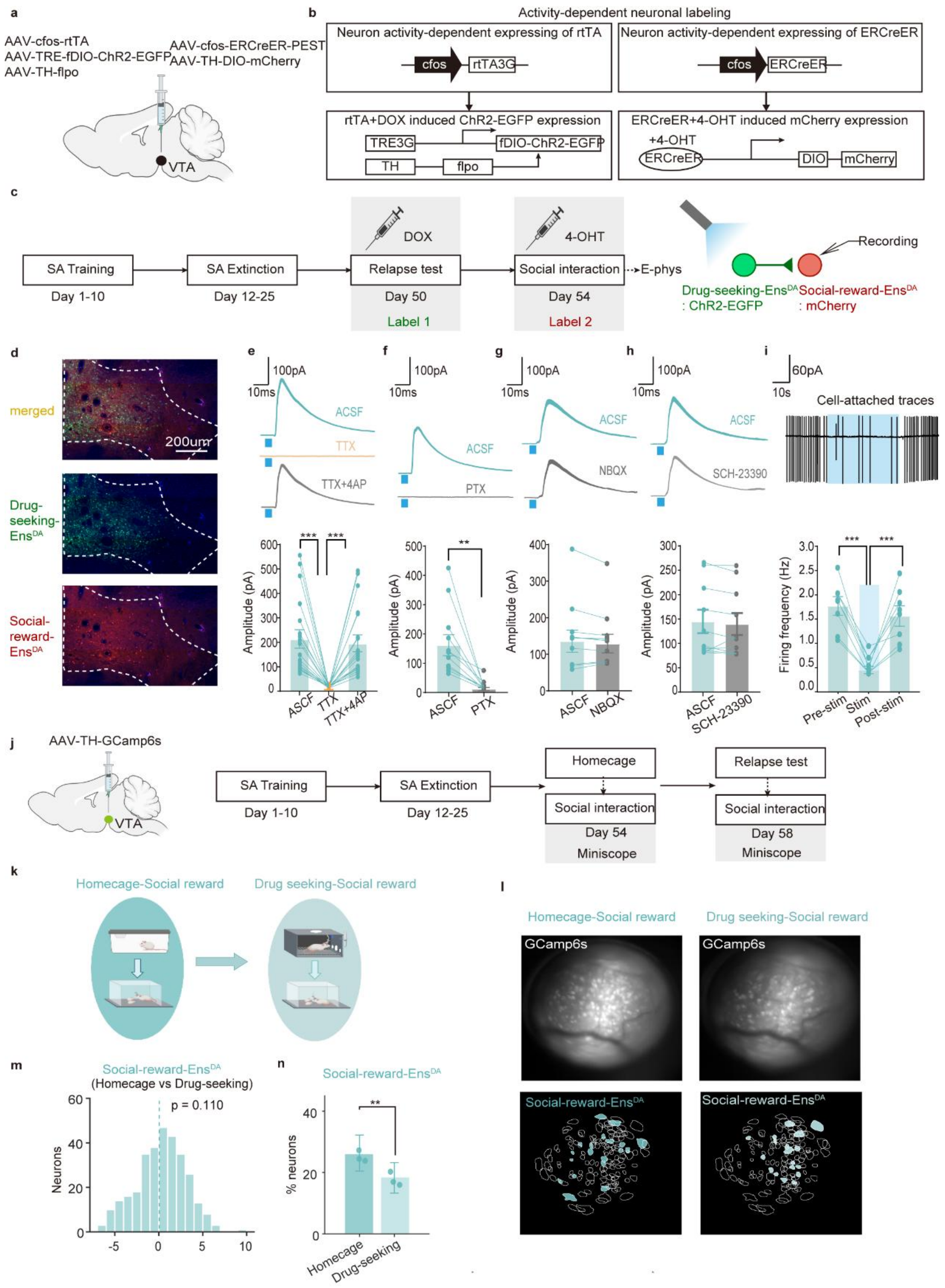

Supplementary Fig.12 | Drug-seeking-Ens^{DA} in the VTA also provides inhibitory input to Social-reward-Ens^{DA}. a, Virus injection strategy. **b**, Activity-dependent neuronal labeling strategy. **c**, Electrophysiology recording procedure. **d**, Representative

histology confirming successful labeling (repeated 4 times independently with similar results). **e**, Representative traces of IPSCs. Light pulses reliably evoked IPSCs (green), TTX completely blocked IPSCs (yellow), and subsequent application of 4-AP in the presence of TTX rescued IPSCs (grey), with amplitudes normalized (*n* = 18 neurons, one-way ANOVA with Bonferroni multiple comparisons test, $F_{(2,34)} = 29.91, p < 0.001$; ACSF vs TTX, $p < 0.001$; TTX vs TTX + 4AP, $p < 0.001$). **f-h**, Evoked IPSCs following application of PTX, NBQX, or SCH-23390; amplitudes normalized (PTX: *n* = 11 neurons, paired two-tailed t test: $t_{(10)} = 4.149, p = 0.002$). **i**, Representative cell-attached traces. Photo-stimulation of Drug-seeking-Ens^{DA} led to a consistent decrease in the spontaneous firing of Social-reward-Ens^{DA} (*n* = 8 neurons, one-way ANOVA with bonferroni multiple comparisons test: $F_{(2,14)} = 33.05$, $p < 0.001$; Pre vs Sti, $p < 0.001$; Sti vs Post, $p < 0.001$). **j**, Schematic of the experimental procedure for comparison of social-reward-responsive cell imaging before and after drug seeking. **k**, Cartoon representing the comparison between social-reward-responsive cell imaging before and after drug seeking. **l**, Example field of view across distinct imaging sessions. **m**, Changes of magnitude in response to social reward before and after exposure to drug seeking (*n* = 319 neurons, two-tailed one-sample t-test, p = 0.110). **n**, Percentage of neurons responsive to social before and after drug seeking (*n* =319 neurons, chi-square test for two proportions, $p = 0.009$). All the data are presented as mean ± SEM. 95% confidence interval was used for all statistical analyses in this figure. Panels a, c, j, k:

Created in BioRender. Xiaoxing, L. (2026) https://BioRender.com/hqs0eg6. $^{*}p < 0.05$, $^{**}p < 0.01$, $^{***}p < 0.001$.

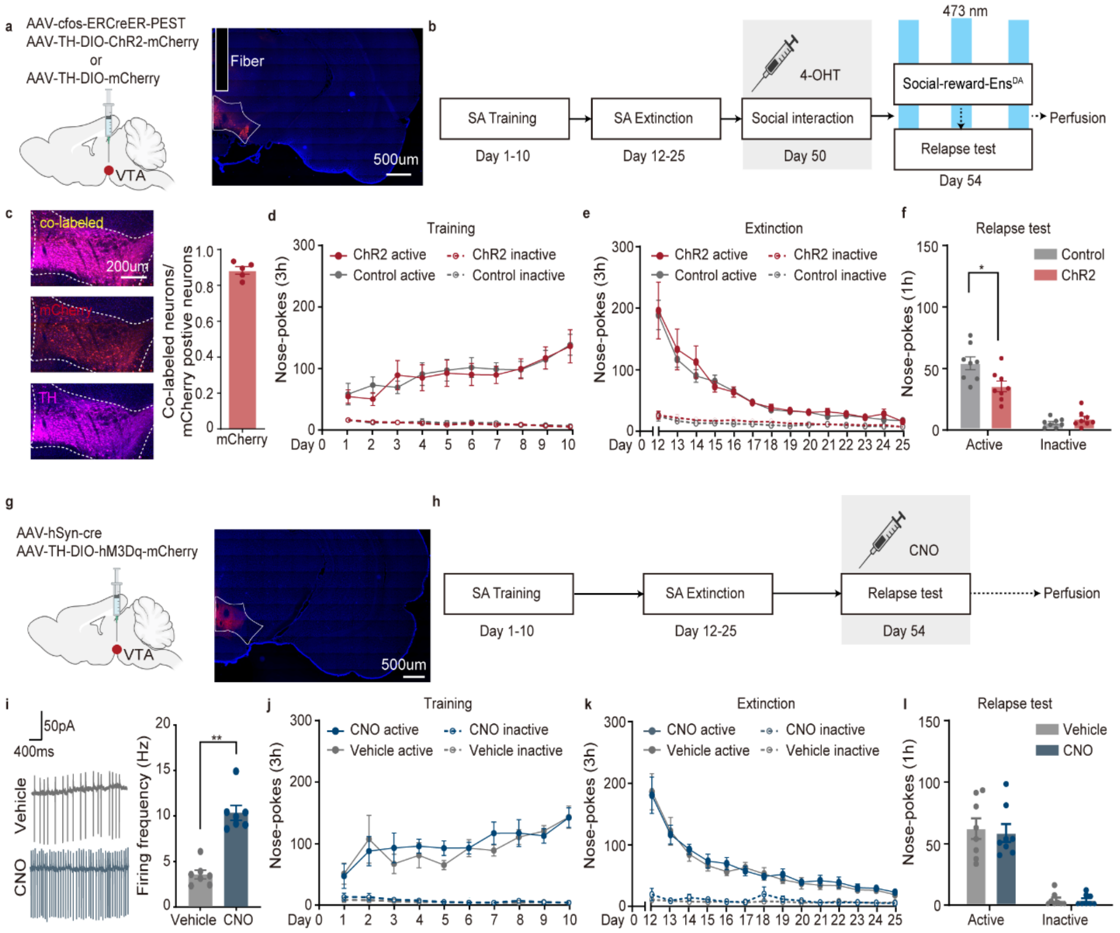

Supplementary Fig.13 | The specific activation of Social-reward-Ens^{DA} in the VTA is critical in the protective effects of social reward on cocaine seeking. a, Virus injection strategy and representative photo of ChR2-mCherry expression (repeated 4 times independently with similar results; scale bar, 500 μm). **b**, Experimental design. **c**, Representative image confirming that the labeled cells are indeed dopaminergic, as verified by TH staining. **d**, Number of nose-pokes during cocaine self-administration training (n = 8 male rats, three-way ANOVA: $F_{(9,6)} = 2.009$, $p = 0.204$; more statistics see Supplementary Table 5G). **e**, Number of nose-pokes during extinction (n = 8 male rats, three-way ANOVA: $F_{(13,2)} = 0.470$, $p = 0.841$; more statistics see Supplementary

Table 5H). **f**, Number of nose-pokes during relapse test (n = 8 male rats, two-way ANOVA: $F_{(1,14)}$ =8.699, p = 0.010, more statistics see Supplementary Table 5I). **g**, Virus injection strategy and representative photo of hM3Dq-mCherry expression (repeated 4 times independently with similar results; scale bar, 500 μm). **h**, Experimental design. **i**, CNO application significantly increased the spontaneous firing rate of hM3Dq-expressing DAergic neurons (n = 7 neurons; unpaired two-tailed t test: $t_{(12)}$ = 7.229, p < 0.001). **j**, Number of nose-pokes during cocaine self-administration training (n = 8 male rats, three-way ANOVA: $F_{(9,6)}$ = 0.785, p = 0.643; more statistics see Supplementary Table 6A). **k**, Number of nose-pokes during extinction (n = 8 male rats, three-way ANOVA: $F_{(13,2)}$ = 0.187, p = 0.980; more statistics see Supplementary Table 6B). **l**, Number of nose-pokes during relapse test (n = 8 male rats, two-way ANOVA: $F_{(1,14)}$ =0.092, p = 0.766; more statistics see Supplementary Table 6C). All the data are presented as mean ± SEM. 95% confidence interval was used for all statistical analyses in this figure. Panels a, b, g, h: Created in BioRender. Xiaoxing, L. (2026) https://BioRender.com/hqs0eg6. $^{*}p < 0.05$, $^{**}p < 0.01$, $^{***}p < 0.001$.

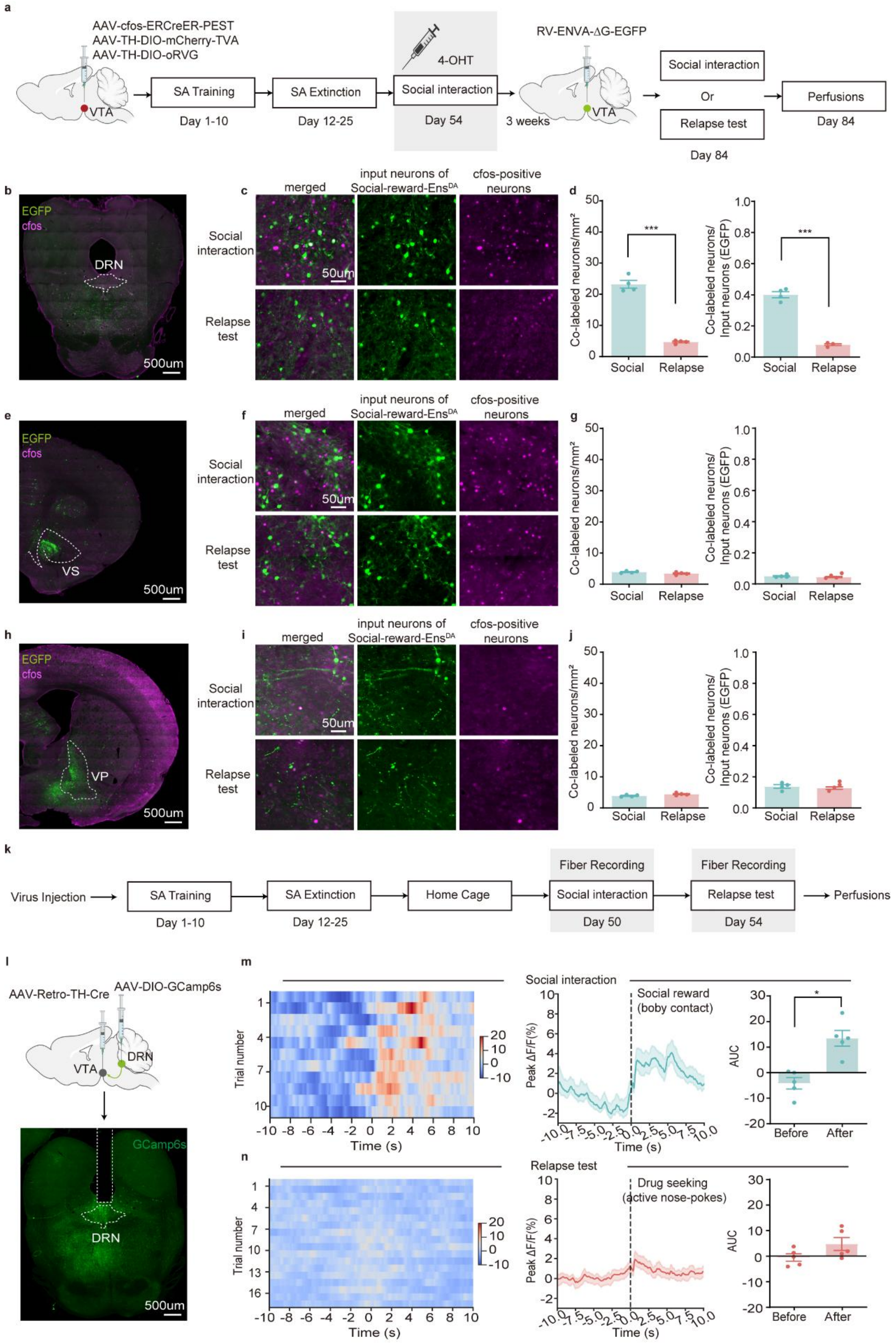
a
AAV-cfos-ERCreER-PEST
AAV-TH-DIO-mCherry-TVA
AAV-TH-DIO-oRVG
VTA
SA Training
Day 1-10
SA Extinction
Day 12-25
4-OHT
Social interaction
Day 54
3 weeks
RV-ENVA-ΔG-EGFP
VTA
Social interaction
Or
Relapse test
Day 84
Perfusions
Day 84
b
EGFP
cfos
DRN
500um
c
merged
input neurons of Social-reward-Ens^DA
cfos-positive neurons
Social interaction
Relapse test
50um
d
Co-labeled neurons/mm²
Co-labeled neurons/ Input neurons (EGFP)
***
Social
Relapse
e
VS
f
g
h
VP
i
j
k
Virus Injection
SA Training
Day 1-10
SA Extinction
Day 12-25
Home Cage
Fiber Recording
Social interaction
Day 50
Fiber Recording
Relapse test
Day 54
Perfusions
l
AAV-Retro-TH-Cre
AAV-DIO-GCamp6s
VTA
DRN
GCamp6s
500um
m
Trial number
Time (s)
Social interaction
Social reward (boby contact)
Peak ΔF/F(%)
AUC
*
Before
After
n
Relapse test
Drug seeking (active nose-pokes)

Supplementary Fig.14 | Social reward significantly activates DRN populations that project to Social-reward-Ens^{DA} in the VTA. a, Schematic of the experimental procedure. **b**, **c**, Representative images of cfos expression and inputs of Social-reward-Ens^{DA} in the DRN at 500 μm (b) and 50 μm (c) magnifications (repeated 4 times independently with similar results). Pink: cfos; Green: EGFP (inputs of Social-reward-responsive DAergic ensembles). **d**, Quantification of co-labeled neuron number (n = 4 male rats, unpaired two-tailed t test: $t_{(6)} = 14.48$, $p < 0.001$) and percentage (n = 4 male rats, unpaired two-tailed t test: $t_{(6)} = 15.85$, $p < 0.001$) in the DRN. **e**, **f**, Representative images of cfos expression and inputs of Social-reward-Ens^{DA} in the VS at 500 μm (e) and 50 μm (f) magnifications (repeated 4 times independently with similar results). **g**, Quantification of co-labeled neuron number and percentage in the VS. **h**, **i**, Representative images of cfos expression and inputs of Social-reward-Ens^{DA} in the VP at 500 μm (h) and 50 μm (i) magnifications (repeated 4 times independently with similar results). **j**, Quantification of co-labeled neuron number and percentage in the VP. **k**, Schematic of the experimental procedure for fiber photometry recording. **l**, Virus injection strategy and the representative image of GCamp6s expression in the DRN (repeated 4 times independently with similar results; scale bar, 500 μm). Green: Gcamp6s. **m**, **n**, Trial-by-trial heatmaps of calcium signals during social reward vs. drug seeking; color scales indicate ΔF/F. Event plots of average calcium signals aligned to behavioral tasks. AUC comparison between baseline (10 s before stimuli) and stimulus (10 s after onset) responses (n = 5 male rats, Social interaction, paired two-tailed t test, $t_{(4)} = 3.460$, $p = 0.026$). All the data are

presented as mean ± SEM. 95% confidence interval was used for all statistical analyses in this figure. Panels a, l: Created in BioRender. Xiaoxing, L. (2026) https://BioRender.com/hqs0eg6. $^{*}p < 0.05$, $^{**}p < 0.01$, $^{***}p < 0.001$.

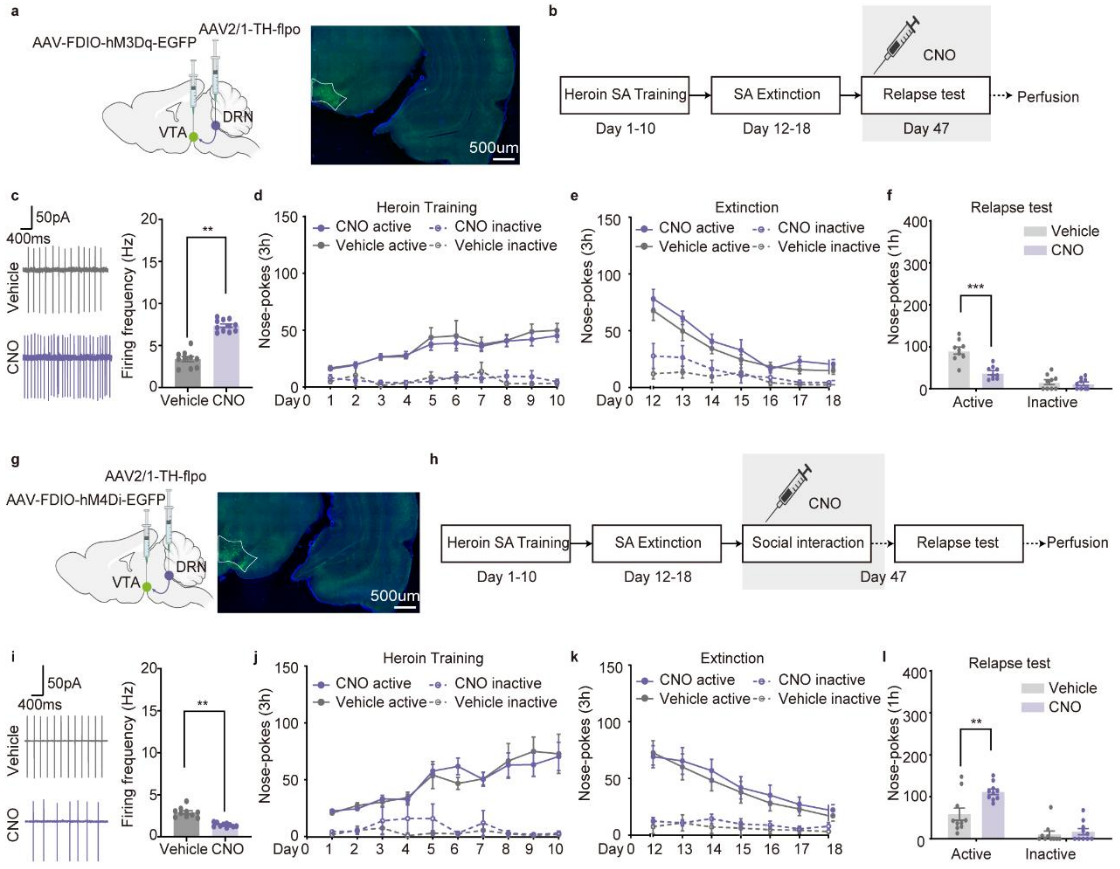

Supplementary Fig.15 | VTA DAergic neurons that receive input from the DRN bidirectionally modulates heroin seeking. **a**, Virus injection strategy and representative photo of hM3Dq-EGFP expression (repeated 4 times independently with similar results; scale bar, 500 μm). **b**, Experimental scheme. **c**, CNO increased spontaneous firing of hM3Dq-expressing VTA DAergic neurons (*n* = 10 neurons, unpaired two-tailed t test: $t_{(18)} = 10.730$, $p < 0.001$). **d**, Number of nose-pokes during heroin self-administration training (*n* = 9 male rats, three-way ANOVA: $F_{(9,8)} = 3.126$, $p = 0.062$; more statistics see Supplementary Table 9A). **e**, Number of nose-pokes during extinction (*n* = 9 male rats, three-way ANOVA: $F_{(6,11)} = 1.668$, $p = 0.219$; more statistics see Supplementary Table 6B). **f**, Number of nose-pokes during relapse test (*n* = 9 male rats, two-way ANOVA with simple effect analysis: $F_{(1,16)} = 16.892$, $p < 0.001$;

more statistics see Supplementary Table 9C). **g**, Virus injection strategy and representative photo of hM4Di-EGFP expression (repeated 4 times independently with similar results; scale bar, 500 μm). **h**, Experimental scheme. **i**, CNO decreased spontaneous firing of hM4Di-expressing VTA DAergic neurons (*n* = 10 neurons, unpaired two-tailed t test: $t_{(18)}$=7.397, p<0.001). **j**, Number of nose-pokes during heroin self-administration training (*n* = 10 male rats, three-way ANOVA: $F_{(9,10)} = 0.687$, $p = 0.708$; more statistics see Supplementary Table 9D). **k**, Number of nose-pokes during extinction (*n* = 10 male rats, three-way ANOVA: $F_{(6,13)} = 0.352$, $p = 0.896$; more statistics see Supplementary Table 9E). **l**, Number of nose-pokes during relapse test (*n* = 10 male rats, two-way ANOVA with simple effect analysis: $F_{(1,18)} = 5.682$, $p = 0.028$; more statistics see Supplementary Table 9F). All the data are presented as mean ± SEM. 95% confidence interval was used for all statistical analyses in this figure. Panels a, b, g, h: Created in BioRender. Xiaoxing, L. (2026) https://BioRender.com/hqs0eg6. $^{*}p < 0.05$, $^{**}p < 0.01$, $^{***}p < 0.001$.

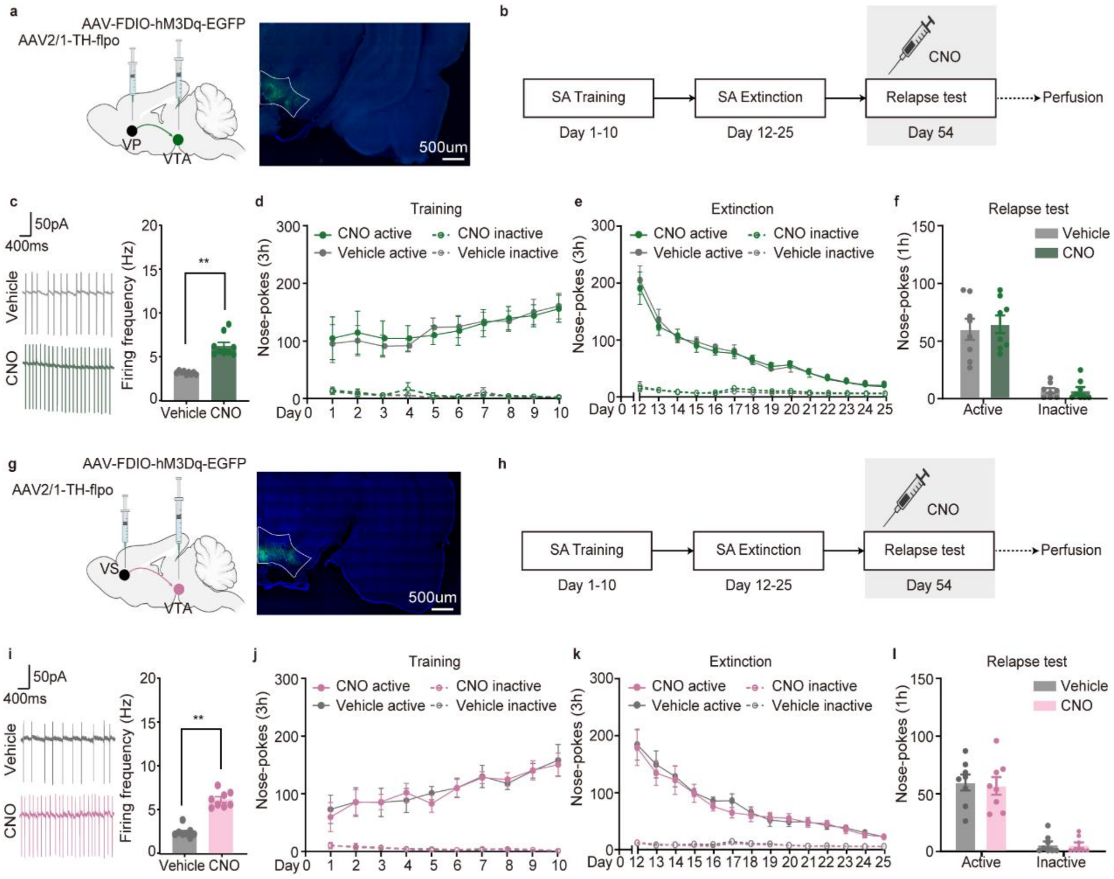

Supplementary Fig.16 | Activation of VTA DAergic neurons that receive input from VP or VS does not affect cocaine seeking. a, Virus injection strategy and representative photo of hM3Dq-EGFP expression (repeated 4 times independently with similar results; scale bar, 500 μm). **b**, Experimental scheme. **c**, CNO increased spontaneous firing of hM3Dq-expressing VTA DAergic neurons (n = 8 neurons, unpaired two-tailed t test: $t_{(14)} = 8.412$, $p < 0.001$). **d**, Number of nose-pokes during cocaine self-administration training (n = 8 male rats, three-way ANOVA: $F_{(9,6)} = 0.466$, $p = 0.854$; more statistics see Supplementary Table 10A). **e**, Number of nose-pokes during extinction (n = 8 male rats, three-way ANOVA: $F_{(13,2)} = 1.456$, $p = 0.480$; more statistics see Supplementary Table 10B). **f**, Number of nose-pokes during relapse test (n = 8 male rats, two-way ANOVA: $F_{(1,14)} = 0.123$, $p = 0.731$; more statistics see

Supplementary Table 10C). **g**, Virus injection strategy and representative photo of hM3Dq-EGFP expression (repeated 4 times independently with similar results; scale bar, 500 μm). **h**, Experimental scheme. **i**, CNO increased spontaneous firing of hM3Dq-expressing VTA DAergic neurons ($n = 8$ neurons, unpaired two-tailed t test: $t_{(14)} = 9.598$, $p < 0.001$). **j**, Number of nose-pokes during cocaine self-administration training ($n = 8$ male rats, three-way ANOVA: $F_{(9,6)} = 0.435$, $p = 0.874$; more statistics see Supplementary Table 10D). **k**, Number of nose-pokes during extinction ($n = 8$ male rats, three-way ANOVA: $F_{(13,2)} = 5.872$, $p = 0.155$; more statistics see Supplementary Table 10E). **l**, Number of nose-pokes during relapse test ($n = 8$ male rats, two-way ANOVA: $F_{(1,14)} = 0.049$, $p = 0.828$; more statistics see Supplementary Table 10F). All the data are presented as mean ± SEM. 95% confidence interval was used for all statistical analyses in this figure. Panels a, b, g, h: Created in BioRender. Xiaoxing, L. (2026) https://BioRender.com/hqs0eg6. $^{*}p < 0.05$, $^{**}p < 0.01$, $^{***}p < 0.001$.

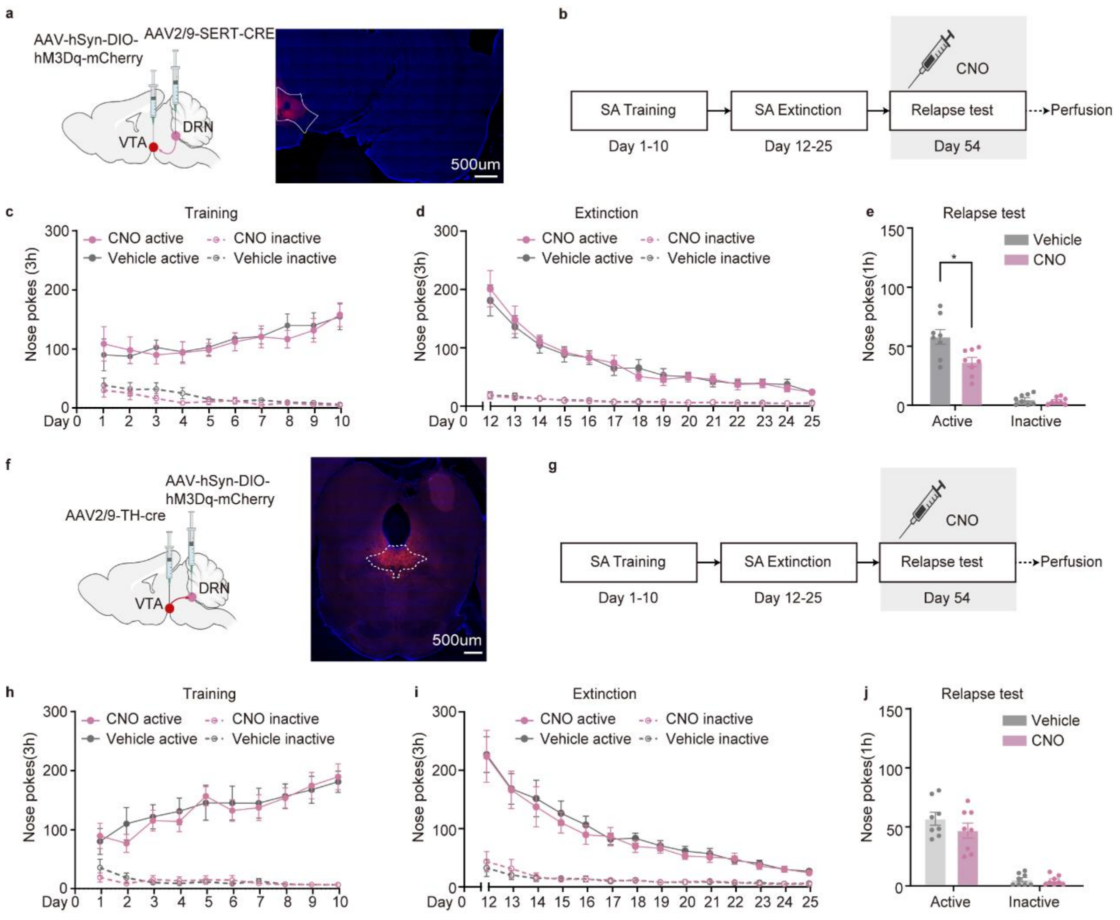

Supplementary Fig.17 | Activation of the serotonergic DRN-VTA but not the dopaminergic VTA-DRN projections mimicked the therapeutic effect of social reward against cocaine seeking. a, Virus injection strategy and representative photo of hM3Dq-mCherry expression in the VTA (repeated 4 times independently with similar results; scale bar, 500 μm). **b**, Experimental scheme. **c**, Number of nose-pokes during cocaine self-administration training (*n* = 8 male rats, three-way ANOVA: $F_{(9,6)}$ = 0.508, p = 0.826; more statistics see Supplementary Table 8G). **d**, Number of nose-pokes during extinction (*n* = 8 male rats, three-way ANOVA: $F_{(13,2)}$ = 2.887, p = 0.286; more statistics see Supplementary Table 8H). **e**, Number of nose-pokes during relapse test (*n* = 8 male rats, two-way ANOVA with simple effect analysis: $F_{(1,14)}$ = 5.803, p = 0.030; more statistics see Supplementary Table 8I). **f**, Virus injection strategy and

representative photo of hM3Dq-mCherry expression in the DRN (repeated 4 times independently with similar results; scale bar, 500 μm). **g**, Experimental scheme. **h**, Number of nose-pokes during cocaine self-administration training (*n* = 8 male rats, three-way ANOVA: $F_{(9,6)} = 0.570$, $p = 0.785$; more statistics see Supplementary Table 8J). **i**, Number of nose-pokes during extinction (*n* = 8 male rats, three-way ANOVA: $F_{(13,2)} = 2.070$, $p = 0.372$; more statistics see Supplementary Table 8K). **j**, Number of nose pokes during relapse test (*n* = 8 male rats, two-way ANOVA with simple effect analysis: $F_{(1,14)} = 1.163$, $p = 0.299$; more statistics see Supplementary Table 8L). All the data are presented as mean ± SEM. 95% confidence interval was used for all statistical analyses in this figure. Panels a, b, f, g: Created in BioRender. Xiaoxing, L. (2026) https://BioRender.com/hqs0eg6. $^{*}p < 0.05$, $^{**}p < 0.01$, $^{***}p < 0.001$.

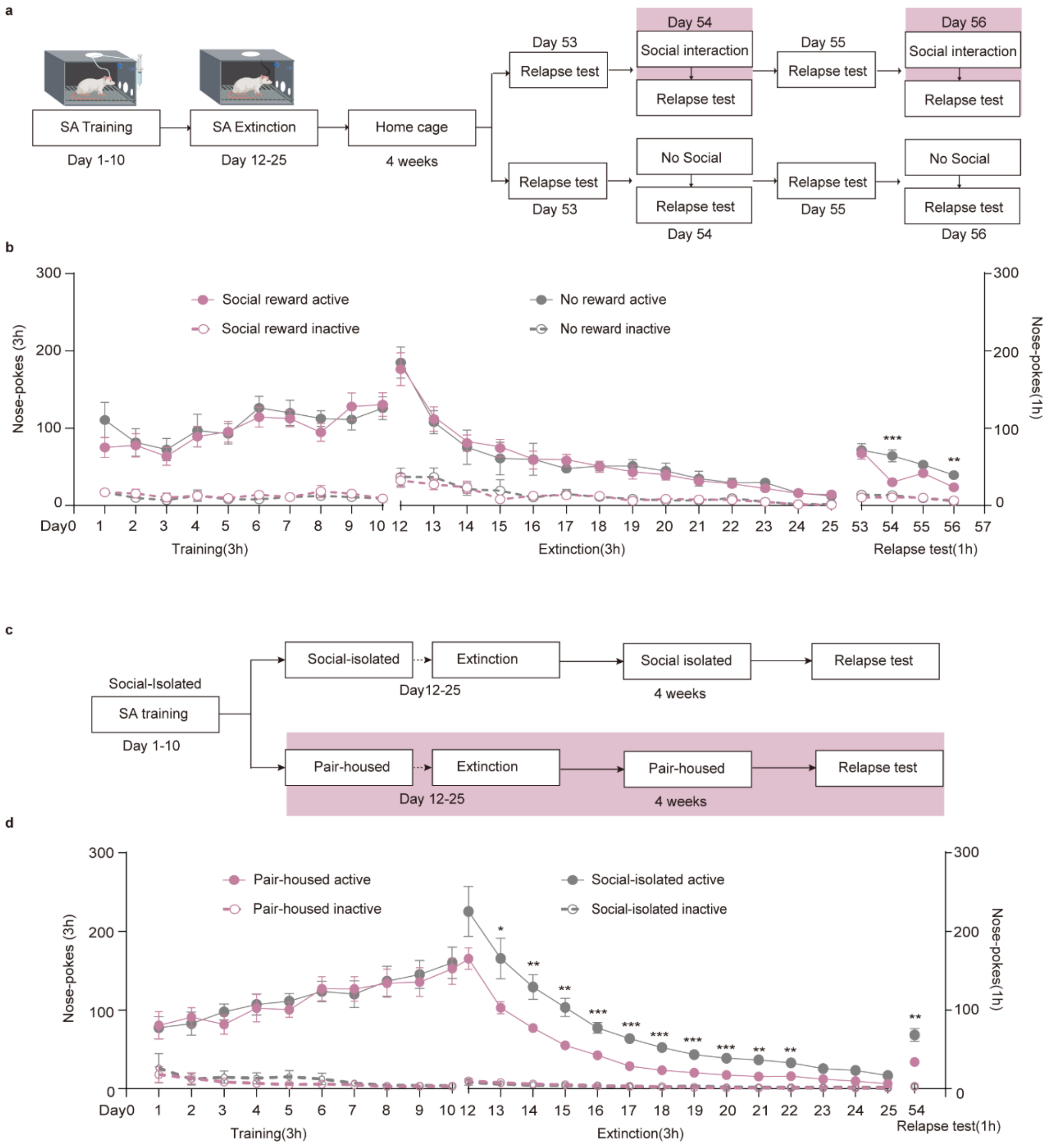

Supplementary Fig.18 | The protective effect of acute social interaction against drug seeking is transient. a, Experimental design to investigate the duration of the suppressive effect of acute social reward on cocaine-seeking behavior (Created in BioRender. Xiaoxing, L. (2026) https://BioRender.com/hqs0eg6). **b**, Number of nose pokes during cocaine self-administration training, extinction, and relapse tests. The protective effect of acute social reward against cocaine seeking was absent 24 h after social interaction (Social reward group, n = 9 male rats; No reward group, n = 8 male

rats; three-way ANOVA with simple simple effect analysis: $F_{(13,3)}$ =36.033, $p < 0.001$; more statistics see Supplementary Table 13A-C). **c**, Experimental design to investigate the effect of housing conditions (pair-housed vs. socially isolated) on cocaine-seeking behavior. **d**, Number of nose-pokes during cocaine self-administration training, extinction and relapse tests. Pair-housed rats exhibited accelerated extinction (Pair-housed group, n = 10 male rats; Social-isolated group, n = 9 male rats; three-way ANOVA with simple simple effect analysis: $F_{(13,5)}$ =4.799, $p = 0.047$; more statistics see Supplementary Table 14B) and reduced cocaine seeking during the relapse test (Pair-housed group, n = 10 male rats; Social-isolated group, n = 9 male rats; two-way ANOVA with simple effect analysis: $F_{(1,17)}$ =14.861, $p = 0.001$; more statistics see Supplementary Table 14C) compared with socially isolated rats. All the data are presented as mean ± SEM. 95% confidence interval was used for all statistical analyses in this figure. $^{*}p < 0.05$, $^{**}p < 0.01$, $^{***}p < 0.001$.

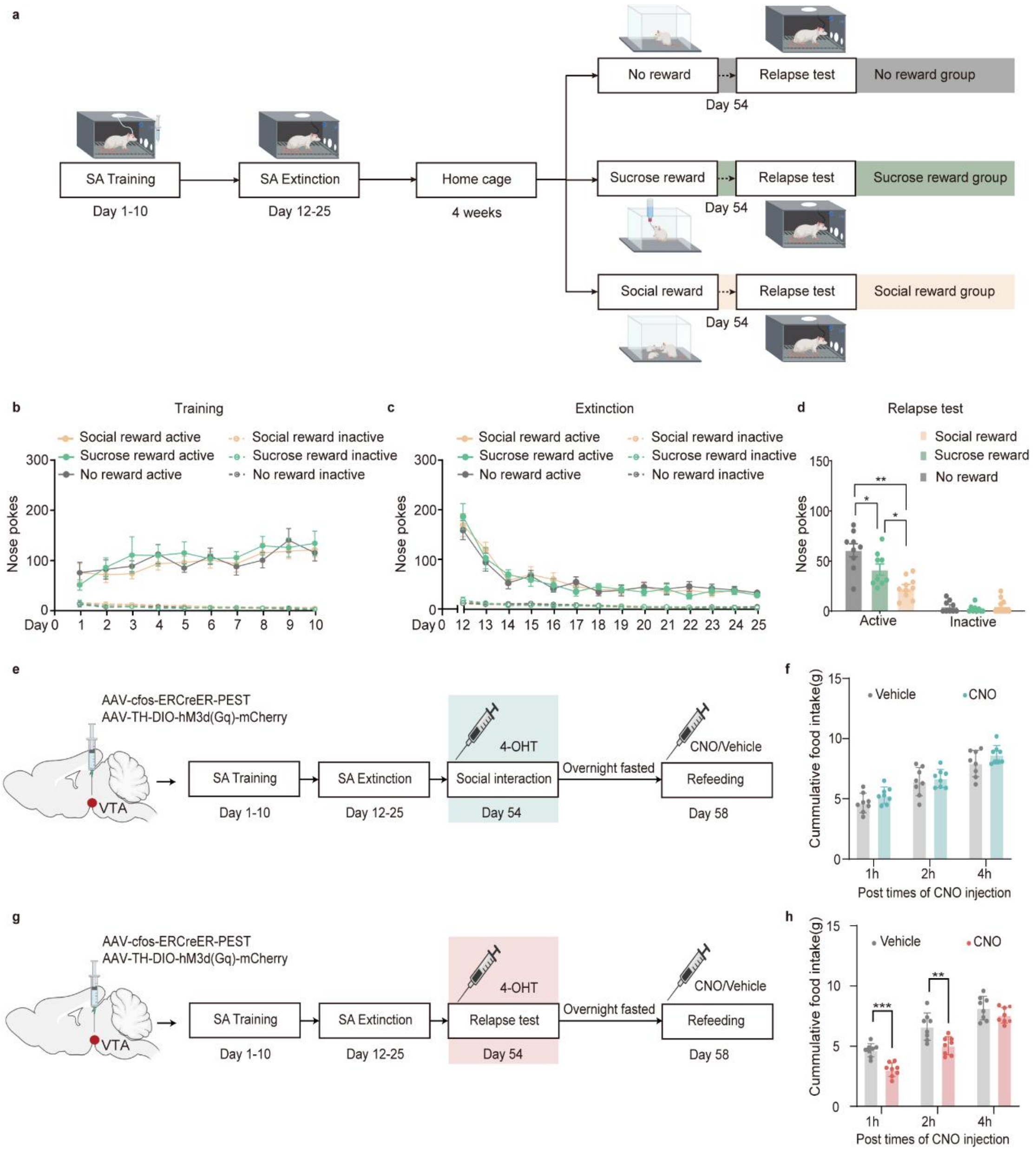

Supplementary Fig.19 | Comparison of therapeutic effects between social and sucrose reward on cocaine seeking. a, Schematic of the experimental design examining the effects of social and sucrose rewards on cocaine seeking. **b**, Number of nose-pokes during cocaine self-administration training (No reward group, n = 9 male rats; Sucrose group, n = 9 male rats; Social reward group, n = 10 male rats; three-way ANOVA: $F_{(9,18)} = 0.673$, $p = 0.723$; more statistics see Supplementary Table 11A). **c**, Number of nose-pokes during extinction (No reward group, n = 9 male rats; Sucrose

group, n = 9 male rats; Social reward group, n = 10 male rats; three-way ANOVA: $F_{(13,14)} = 1.925$, $p = 0.119$; more statistics see Supplementary Table 11B). **d**, Number of nose-pokes during the cocaine relapse test. Sucrose reward reduced cue-induced active nose-pokes, although its therapeutic efficacy was lower than that of social reward (two-way ANOVA with simple effect analysis: $F_{(2,25)} = 15.988$, $p < 0.001$; more statistics see Supplementary Table 11C). **e**, Schematic of the experimental design for refeeding following activation of Social-reward-Ens^{DA}. **f**, Comparison of cumulative food intake in fasted rats. Rats received i.p. injections of CNO or vehicle 30 min prior to being given free access to food. **g**, Schematic of the experimental design for refeeding after activation of Drug-seeking-Ens^{DA}. **h**, Comparison of cumulative food intake in fasted rats. Rats received i.p. injections of CNO or vehicle 30 min prior to being given free access to food. Fasted rats administered CNO consumed significantly less food than those administered vehicle during the first 1 and 2 h of refeeding (n = 8 male rats, two-way ANOVA with simple effect analysis, $F_{(2,13)} = 5.545$, $p = 0.018$; more statistics see Supplementary Table 15B). All the data are presented as mean ± SEM. 95% confidence interval was used for all statistical analyses in this figure. Panels a, e, g: Created in BioRender. Xiaoxing, L. (2026) https://BioRender.com/hqs0eg6. $^{*}p < 0.05$, $^{**}p < 0.01$, $^{***}p < 0.001$.

Supplementary Tables

Supplementary Table 1. Social reward suppresses drug relapse

Figure number	Factor name	F-value	*p*-value
A. SA training—Social suppresses cocaine relapse	Nose-pokes (three-way ANOVA):		
	Nose-poke(within)	$F_{(1, 18)}$ =122.645	<0.001***
	Days (within)	$F_{(9, 10)}$ =2.354	0.099
	Treatment (between)	$F_{(1, 18)}$ =0.202	0.659
	Nose-poke * treatment	$F_{(1, 18)}$ =0.902	0.355
	Days* treatment	$F_{(9, 10)}$ =2.054	0.139
	Days * nose-poke	$F_{(9, 10)}$ =8.680	0.001**
	Days * nose-poke * treatment	$F_{(9, 10)}$ =2.799	0.062
B. SA extinction—Social suppresses cocaine relapse	Nose-pokes (three-way ANOVA):		
	Nose-poke(within)	$F_{(1, 18)}$ =79.724	<0.001***
	Days (within)	$F_{(13, 6)}$ =13.334	0.002**
	Treatment (between)	$F_{(1, 18)}$ =0.017	0.898
	Nose-poke * treatment	$F_{(1, 18)}$ =0.706	0.412
	Days* treatment	$F_{(13, 6)}$ =4.235	0.043*
	Nose-poke * days	$F_{(13, 6)}$ =11.219	0.004**
	Nose-poke * days* treatment	$F_{(13, 6)}$ =1.223	0.425
C. Relapse test—Social suppresses cocaine relapse	Nose-pokes (two-way ANOVA):		
	Nose-poke(within)	$F_{(1, 18)}$ =44.178	<0.001***
	Treatment (between)	$F_{(1, 18)}$ =11.850	0.003**
	Nose-poke * treatment	$F_{(1, 18)}$ =5.857	0.026*
	Simple effect analysis：		
	Active	$F_{(1, 18)}$ =10.880	0.004**
	Inactive	$F_{(1, 18)}$ =1.018	0.326
D. SA training—Social suppresses heroin relapse	Nose-pokes (three-way ANOVA):		
	Nose-poke(within)	$F_{(1, 16)}$ =67.829	<0.001***
	Days (within)	$F_{(9, 8)}$ =3.801	0.037*
	Treatment (between)	$F_{(1, 16)}$ =0.239	0.632
	Nose-poke * treatment	$F_{(1, 16)}$ =0.009	0.928

	Days* treatment<br>Days * nose-poke<br>Days * nose-poke * treatment	$F_{(9,\ 8)}$ =1.715<br>$F_{(9, 8)}$ =5.829<br>$F_{(9,\ 8)}$ =1.582	0.230<br>0.010*<br>0.265
E. SA extinction —Social suppresses heroin relapse	Nose-pokes (three-way ANOVA):<br>Nose-poke(within)<br>Days (within)<br>Treatment (between)<br>Nose-poke * treatment<br>Days* treatment<br>Nose-poke * days<br>Nose-poke * days* treatment	 $F_{(1, 16)}$ =68.412<br>$F_{(6, 11)}$ =8.327<br>$F_{(1, 16)}$ =0.125<br>$F_{(1, 16)}$ =0.411<br>$F_{(6, 11)}$ =0.300<br>$F_{(6, 11)}$ =12.313<br>$F_{(6, 11)}$ =2.739	 <0.001***<br>0.001**<br>0.729<br>0.531<br>0.924<br><0.001***<br>0.070
F. Relapse test—Social suppresses heroin relapse	Nose-pokes (two-way ANOVA):<br>Nose-poke(within)<br>Treatment (between)<br>Nose-poke * treatment<br><br>Simple effect analysis：<br>Active<br>Inactive	 $F_{(1, 16)}$ =78.247<br>$F_{(1, 16)}$ =14.533<br>$F_{(1, 16)}$ =16.919<br><br><br>$F_{(1, 16)}$ =21.926<br>$F_{(1, 16)}$ =1.293	 <0.001***<br>0.002**<br><0.001***<br><br><br><0.001***<br>0.272

Supplementary Table 2. Both toy rat interaction and anesthetized rat interaction have no influence on cocaine relapse

Figure number	Factor name	F-value	*p*-value
A. SA training—Toy interaction	Nose-pokes (three-way ANOVA):		
	Nose-poke(within)	$F_{(1, 14)}$ =124.354	<0.001***
	Days (within)	$F_{(9, 6)}$ =4.160	0.048*
	Treatment (between)	$F_{(1, 14)}$ =0.018	0.896
	Nose-poke * treatment	$F_{(1, 14)}$ =0.073	0.792
	Days* treatment	$F_{(9, 6)}$ =0.400	0.895
	Days * nose-poke	$F_{(9, 6)}$ =8.842	0.008**
	Days * nose-poke * treatment	$F_{(9, 6)}$ =0.397	0.897
B. SA extinction—Toy interaction	Nose-pokes (three-way ANOVA):		
	Nose-poke(within)	$F_{(1, 14)}$ =187.879	<0.001***
	Days (within)	$F_{(13, 2)}$ =168.912	0.006**
	Treatment (between)	$F_{(1, 14)}$ =1.116	0.309
	Nose-poke * treatment	$F_{(1, 14)}$ =0.002	0.970
	Days* treatment	$F_{(13, 2)}$ =0.783	0.688
	Nose-poke * days	$F_{(13, 2)}$ =6.464	0.142
	Nose-poke * days* treatment	$F_{(13, 2)}$ =0.962	0.619
C. Relapse test—Toy interaction	Nose-pokes (two-way ANOVA):		
	Nose-poke(within)	$F_{(1, 14)}$ =136.471	<0.001***
	Treatment (between)	$F_{(1, 14)}$ =3.268	0.092
	Nose-poke * treatment	$F_{(1, 14)}$ =2.168	0.163
D. SA training—Anesthetized rat interaction	Nose-pokes (three-way ANOVA):		
	Nose-poke(within)	$F_{(1, 14)}$ =2.541	0.133
	Days (within)	$F_{(9, 6)}$ =17.509	0.001**
	Treatment (between)	$F_{(1, 14)}$ =0.000	0.988
	Nose-poke * treatment	$F_{(1, 14)}$ =0.284	0.602
	Days* treatment	$F_{(9, 6)}$ =0.447	0.867
	Days * nose-poke	$F_{(9, 6)}$ =1.230	0.414
	Days * nose-poke * treatment	$F_{(9, 6)}$ =0.284	0.955
E. SA extinction—	Nose-pokes (three-way ANOVA):		
	Nose-poke(within)	$F_{(1, 14)}$ =32.506	<0.001***

Anesthetized rat interaction	Days (within)<br>Treatment (between)<br>Nose-poke * treatment<br>Days* treatment<br>Nose-poke * days<br>Nose-poke * days* treatment	$F_{(13, 2)}$ =12.644<br>$F_{(1, 14)}$ =0.001<br>$F_{(1, 14)}$ =0.224<br>$F_{(13, 2)}$ =2.186<br>$F_{(13, 2)}$ =4.161<br>$F_{(13, 2)}$ =1.011	0.076<br>0.974<br>0.643<br>0.357<br>0.210<br>0.602
F. Relapse test—Anesthetized rat interaction	Nose-pokes (two-way ANOVA):<br>Nose-poke(within)<br>Treatment (between)<br>Nose-poke * treatment	 $F_{(1, 14)}$ =158.873<br>$F_{(1, 14)}$ =2.484<br>$F_{(1, 14)}$ =2.339	 <0.001***<br>0.137<br>0.148

Supplementary Table 3. Social reward inhibited the activation of DAergic neurons in the VTA caused by cocaine relapse

Figure number	Factor name	F-value	*p*-value
A. SA training—Fiber photometry recording	Nose-pokes (three-way ANOVA):		
	Nose-poke(within)	$F_{(1, 18)}$ =1.685	0.211
	Days (within)	$F_{(9, 10)}$ =15.671	<0.001***
	Treatment (between)	$F_{(1, 18)}$ =0.078	0.784
	Nose-poke * treatment	$F_{(1, 18)}$ =0.027	0.870
	Days* treatment	$F_{(9, 10)}$ =1.445	0.287
	Days * nose-poke	$F_{(9, 10)}$ =2.204	0.117
	Days * nose-poke * treatment	$F_{(9, 10)}$ =1.157	0.409
B. SA extinction—Fiber photometry recording	Nose-pokes (three-way ANOVA):		
	Nose-poke(within)	$F_{(1, 18)}$ =69.226	<0.001***
	Days (within)	$F_{(13, 6)}$ =23.811	<0.001***
	Treatment (between)	$F_{(1, 18)}$ =0.538	0.473
	Nose-poke * treatment	$F_{(1, 18)}$ =0.645	0.432
	Days* treatment	$F_{(13, 6)}$ =0.694	0.728
	Nose-poke * days	$F_{(13, 6)}$ =2.360	0.150
	Nose-poke * days* treatment	$F_{(13, 6)}$ =0.409	0.917

Supplementary Table 4. Separate DA subpopulations in the VTA response to social reward and cocaine relapse

Figure number	Factor name	F-value	*p*-value
A. SA training—TRAP and cfos	Nose-pokes (three-way ANOVA):		
	Nose-poke(within)	$F_{(1, 14)} = 1.236$	0.285
	Days (within)	$F_{(9, 6)} = 11.343$	0.004**
	Treatment (between)	$F_{(3, 14)} = 0.057$	0.982
	Nose-poke * treatment	$F_{(3, 14)} = 0.988$	0.427
	Days* treatment	$F_{(9, 8)} = 13.342$	0.004**
	Days * nose-poke	$F_{(9, 6)} = 1.088$	0.477
	Days * nose-poke * treatment	$F_{(9, 8)} = 2.116$	0.152
B. SA extinction—TRAP and cfos	Nose-pokes (three-way ANOVA):		
	Nose-poke(within)	$F_{(1, 14)} = 44.852$	<0.001***
	Days (within)	$F_{(13, 2)} = 16.451$	0.059
	Treatment (between)	$F_{(3, 14)} = 0.035$	0.991
	Nose-poke * treatment	$F_{(3, 14)} = 0.955$	0.441
	Days* treatment	$F_{(13, 4)} = 21.605$	0.005**
	Nose-poke * days	$F_{(13, 2)} = 29.449$	0.033*
	Nose-poke * days* treatment	$F_{(13, 4)} = 164.483$	<0.001***

Supplementary Table 5. Social-reward-Ens^{DA} in the VTA bidirectionally regulates cocaine relapse

Figure number	Factor name	F-value	*p*-value
A. SA training—activation of Social-reward-Ens^{DA}	Nose-pokes (three-way ANOVA):		
	Nose-poke(within)	$F_{(1, 16)}$ =109.138	<0.001***
	Days (within)	$F_{(9, 8)}$ =15.469	<0.001***
	Treatment (between)	$F_{(1, 16)}$ =0.209	0.654
	Nose-poke * treatment	$F_{(1, 16)}$ =0.004	0.953
	Days* treatment	$F_{(9, 8)}$ =1.444	0.308
	Days * nose-poke	$F_{(9, 8)}$ =10.657	0.001**
	Days * nose-poke * treatment	$F_{(9, 8)}$ =0.274	0.965
B. SA extinction—activation of Social-reward-Ens^{DA}	Nose-pokes (three-way ANOVA):		
	Nose-poke(within)	$F_{(1, 16)}$ =139.740	<0.001***
	Days (within)	$F_{(13, 4)}$ =32.620	0.002**
	Treatment (between)	$F_{(1, 16)}$ =0.001	0.982
	Nose-poke * treatment	$F_{(1, 16)}$ =0.561	0.465
	Days* treatment	$F_{(13, 4)}$ =0.634	0.761
	Nose-poke * days	$F_{(13, 4)}$ =16.524	0.008**
	Nose-poke * days* treatment	$F_{(13, 4)}$ =0.534	0.825
C. Relapse test—activation of Social-reward-Ens^{DA}	Nose-pokes (two-way ANOVA):		
	Nose-poke(within)	$F_{(1, 16)}$ =132.172	<0.001***
	Treatment (between)	$F_{(1, 16)}$ =7.893	0.013*
	Nose-poke * treatment	$F_{(1, 16)}$ =12.222	0.003**
	Simple effect analysis：		
	Active	$F_{(1, 16)}$ =10.098	0.006**
	Inactive	$F_{(1, 16)}$ =0.058	0.812
D. SA training—inhibition of Social-reward-Ens^{DA}	Nose-pokes (three-way ANOVA):		
	Nose-poke(within)	$F_{(1, 20)}$ =159.604	<0.001***
	Days (within)	$F_{(9, 12)}$ =6.023	0.003**
	Treatment (between)	$F_{(1, 20)}$ =0.004	0.949
	Nose-poke * treatment	$F_{(1, 20)}$ =0.001	0.977
	Days* treatment	$F_{(9, 12)}$ =0.939	0.527
	Days * nose-poke	$F_{(9, 12)}$ =32.414	<0.001***
	Days * nose-poke * treatment	$F_{(9, 12)}$ =0.464	0.873

E. SA extinction training—inhibition of Social-reward-Ens[DA]	Nose-pokes (three-way ANOVA):		
	Nose-poke(within)	$F_{(1, 20)}$ =137.461	<0.001***
	Days (within)	$F_{(13, 8)}$ =17.347	0.001**
	Treatment (between)	$F_{(1, 20)}$ =0.046	0.832
	Nose-poke * treatment	$F_{(1, 20)}$ =0.000	0.986
	Days* treatment	$F_{(13, 8)}$ =0.913	0.576
	Nose-poke * days	$F_{(13, 8)}$ =10.628	0.001**
	Nose-poke * days* treatment	$F_{(13, 8)}$ =0.988	0.528
F. Relapse test—inhibition of Social-reward-Ens[DA]	Nose-pokes (two-way ANOVA):		
	Nose-poke(within)	$F_{(1, 20)}$ =144.649	<0.001***
	Treatment (between)	$F_{(1, 20)}$ =5.086	0.035*
	Nose-poke * treatment	$F_{(1, 20)}$ =6.642	0.018*
	Simple effect analysis：		
	Active	$F_{(1, 20)}$ =5.859	0.025*
	Inactive	$F_{(1, 20)}$ =0.012	0.915
G. SA training—opto-activation of Social-reward-Ens[DA]	Nose-pokes (three-way ANOVA):		
	Nose-poke(within)	$F_{(1, 14)}$ =6.374	0.024*
	Days (within)	$F_{(9, 6)}$ =6.422	0.017*
	Treatment (between)	$F_{(1, 14)}$ =0.044	0.837
	Nose-poke * treatment	$F_{(1, 14)}$ =2.114	0.168
	Days* treatment	$F_{(9, 6)}$ =0.383	0.905
	Days * nose-poke	$F_{(9, 6)}$ =1.978	0.210
	Days * nose-poke * treatment	$F_{(9, 6)}$ =2.009	0.204
H. SA extinction—opto-activation of Social-reward-Ens[DA]	Nose-pokes (three-way ANOVA):		
	Nose-poke(within)	$F_{(1, 14)}$ =98.252	<0.001***
	Days (within)	$F_{(13, 2)}$ =11.637	0.082
	Treatment (between)	$F_{(1, 14)}$ =0.356	0.560
	Nose-poke * treatment	$F_{(1, 14)}$ =0.020	0.889
	Days* treatment	$F_{(13, 2)}$ =0.907	0.639
	Nose-poke * days	$F_{(13, 2)}$ =5.673	0.160
	Nose-poke * days* treatment	$F_{(13, 2)}$ =0.470	0.841
I. Relapse test—opto-activation	Nose-pokes (two-way ANOVA):		
	Nose-poke(within)	$F_{(1, 14)}$ =102.020	<0.001***

of Social-reward-Ens^{DA}	Treatment (between)	$F_{(1, 14)}=4.932$	0.043*
	Nose-poke * treatment	$F_{(1, 14)}=8.699$	0.010*
	Simple effect analysis：		
	Active	$F_{(1, 14)}=7.722$	0.015*
	Inactive	$F_{(1, 16)}=1.518$	0.238

Supplementary Table 6. Random activation of DAergic neurons in the VTA has no effect on cocaine relapse

Figure number	Factor name	F-value	p-value
A. SA training—random activation of DAergic neurons	Nose-pokes (three-way ANOVA):		
	Nose-poke(within)	$F_{(1, 14)}=150.461$	<0.001***
	Days (within)	$F_{(9, 6)}=10.823$	0.004**
	Treatment (between)	$F_{(1, 14)}=0.473$	0.503
	Nose-poke * treatment	$F_{(1, 14)}=0.132$	0.722
	Days* treatment	$F_{(9, 6)}=0.445$	0.868
	Days * nose-poke	$F_{(9, 6)}=17.840$	0.001**
	Days * nose-poke * treatment	$F_{(9, 6)}=0.785$	0.643
B. SA extinction—random activation of DAergic neurons	Nose-pokes (three-way ANOVA):		
	Nose-poke(within)	$F_{(1, 14)}=133.831$	<0.001***
	Days (within)	$F_{(13, 2)}=7.313$	0.127
	Treatment (between)	$F_{(1, 14)}=0.528$	0.480
	Nose-poke * treatment	$F_{(1, 14)}=0.003$	0.960
	Days* treatment	$F_{(13, 2)}=0.048$	1.000
	Nose-poke * days	$F_{(13, 2)}=5.944$	0.153
	Nose-poke * days* treatment	$F_{(13, 2)}=0.187$	0.980
C. Relapse test—random activation of DAergic neurons	Nose-pokes (two-way ANOVA):		
	Nose-poke(within)	$F_{(1, 14)}=99.824$	<0.001***
	Treatment (between)	$F_{(1, 14)}=0.104$	0.752
	Nose-poke * treatment	$F_{(1, 14)}=0.092$	0.766

Supplementary Table 7. Social-reward-Ens^{DA} and Drug-seeking-Ens^{DA} in the VTA establish different connections with the brain regions

Figure number	Factor name	F-value	*p*-value
A. SA training—Retro	Nose-pokes (three-way ANOVA):		
	Nose-poke(within)	$F_{(1, 14)}$ =189.373	<0.001***
	Days (within)	$F_{(9, 6)}$ =4.231	0.047*
	Treatment (between)	$F_{(1, 14)}$ =1.031	0.327
	Nose-poke * treatment	$F_{(1, 14)}$ =0.144	0.710
	Days* treatment	$F_{(9, 6)}$ =1.394	0.354
	Days * nose-poke	$F_{(9, 6)}$ =7.542	0.012*
	Days * nose-poke * treatment	$F_{(9, 6)}$ =1.892	0.225
B. SA extinction—Retro	Nose-pokes (three-way ANOVA):		
	Nose-poke(within)	$F_{(1, 14)}$ =82.020	<0.001***
	Days (within)	$F_{(13, 2)}$ =21.659	0.045*
	Treatment (between)	$F_{(1, 14)}$ =0.100	0.988
	Nose-poke * treatment	$F_{(1, 14)}$ =3.679	0.076
	Days* treatment	$F_{(13, 2)}$ =0.745	0.705
	Nose-poke * days	$F_{(13, 2)}$ =6.874	0.134
	Nose-poke * days* treatment	$F_{(13, 2)}$ =1.614	0.447

Supplementary Table 8. DRN-VTA^{DA} bidirectionally regulates cocaine relapse

Figure number	Factor name	F-value	*p*-value
A. SA training—activation of DRN-VTA^{DA}	Nose-pokes (three-way ANOVA):		
	Nose-poke(within)	$F_{(1, 14)}=0.091$	0.768
	Days (within)	$F_{(9, 6)}=38.241$	<0.001***
	Treatment (between)	$F_{(1, 14)}=0.089$	0.769
	Nose-poke * treatment	$F_{(1, 14)}=0.737$	0.405
	Days* treatment	$F_{(9, 6)}=0.839$	0.610
	Days * nose-poke	$F_{(9, 6)}=2.537$	0.135
	Days * nose-poke * treatment	$F_{(9, 6)}=0.843$	0.607
B. SA extinction—activation of DRN-VTA^{DA}	Nose-pokes (three-way ANOVA):		
	Nose-poke(within)	$F_{(1, 14)}=28.142$	<0.001***
	Days (within)	$F_{(13, 2)}=10.743$	0.088
	Treatment (between)	$F_{(1, 14)}=0.947$	0.347
	Nose-poke * treatment	$F_{(1, 14)}=0.430$	0.523
	Days* treatment	$F_{(13, 2)}=0.301$	0.932
	Nose-poke * days	$F_{(13, 2)}=4.011$	0.217
	Nose-poke * days* treatment	$F_{(13, 2)}=6.508$	0.141
C. Relapse test—activation of DRN-VTA^{DA}	Nose-pokes (two-way ANOVA):		
	Nose-poke(within)	$F_{(1, 14)}=67.002$	<0.001***
	Treatment (between)	$F_{(1, 14)}=5.540$	0.034
	Nose-poke * treatment	$F_{(1, 14)}=8.673$	0.011*
	Simple effect analysis：		
	Active	$F_{(1, 14)}=9.450$	0.008**
	Inactive	$F_{(1, 14)}=1.155$	0.301
D. SA training—inhibition of DRN-VTA^{DA}	Nose-pokes (three-way ANOVA):		
	Nose-poke(within)	$F_{(1, 14)}=6.883$	0.020*
	Days (within)	$F_{(9, 6)}=11.256$	0.004**
	Treatment (between)	$F_{(1, 14)}=0.076$	0.787
	Nose-poke * treatment	$F_{(1, 14)}=0.000$	0.987
	Days* treatment	$F_{(9, 6)}=1.429$	0.342
	Days * nose-poke	$F_{(9, 6)}=1.701$	0.266
	Days * nose-poke * treatment	$F_{(9, 6)}=1.439$	0.339

E. SA extinction—inhibition of DRN-VTA^{DA}	Nose-pokes (three-way ANOVA):<br>Nose-poke(within)<br>Days (within)<br>Treatment (between)<br>Nose-poke * treatment<br>Days* treatment<br>Nose-poke * days<br>Nose-poke * days* treatment	 $F_{(1, 14)}$ =70.854<br>$F_{(13, 2)}$ =14.317<br>$F_{(1, 14)}$ =0.029<br>$F_{(1, 14)}$ =1.368<br>$F_{(13, 2)}$ =2.042<br>$F_{(13, 2)}$ =3.222<br>$F_{(13, 2)}$ =1.143	 <0.001***<br>0.067<br>0.868<br>0.262<br>0.376<br>0.262<br>0.560
F. Relapse test—inhibition of DRN-VTA^{DA}	Nose-pokes (two-way ANOVA):<br>Nose-poke(within)<br>Treatment (between)<br>Nose-poke * treatment<br><br>Simple effect analysis：<br>Active<br>Inactive	 $F_{(1, 14)}$ =116.156<br>$F_{(1, 14)}$ =19.025<br>$F_{(1, 14)}$ =15.180<br><br><br>$F_{(1, 14)}$ =19.748<br>$F_{(1, 14)}$ =0.137	 <0.001***<br>0.001**<br>0.002**<br><br><br><0.001***<br>0.717
G. SA training—activation of the serotonergic DRN-VTA projections	Nose-pokes (three-way ANOVA):<br>Nose-poke(within)<br>Days (within)<br>Treatment (between)<br>Nose-poke * treatment<br>Days* treatment<br>Days * nose-poke<br>Days * nose-poke * treatment	 $F_{(1, 14)}$ =0.468<br>$F_{(9, 6)}$ =14.382<br>$F_{(1, 14)}$ =0.203<br>$F_{(1, 14)}$ =0.015<br>$F_{(9, 6)}$ =1.934<br>$F_{(9, 6)}$ =1.876<br>$F_{(9, 6)}$ =0.508	 0.505<br>0.002**<br>0.659<br>0.903<br>0.218<br>0.229<br>0.826
H. SA extinction—activation of the serotonergic DRN-VTA projections	Nose-pokes (three-way ANOVA):<br>Nose-poke(within)<br>Days (within)<br>Treatment (between)<br>Nose-poke * treatment<br>Days* treatment<br>Nose-poke * days<br>Nose-poke * days* treatment	 $F_{(1, 14)}$ =42.883<br>$F_{(13, 2)}$ =11.368<br>$F_{(1, 14)}$ =0.008<br>$F_{(1, 14)}$ =0.150<br>$F_{(13, 2)}$ =0.390<br>$F_{(13, 2)}$ =27.378<br>$F_{(13, 2)}$ =2.887	 <0.001***<br>0.084<br>0.929<br>0.704<br>0.885<br>0.036*<br>0.286
I. Relapse test—activation of the	Nose-pokes (two-way ANOVA):<br>Nose-poke(within)	 $F_{(1, 14)}$ =107.931	 <0.001***

serotonergic DRN-VTA projections	Treatment (between)<br>Nose-poke * treatment<br><br>Simple effect analysis:<br>Active<br>Inactive	$F_{(1, 14)}$ =10.889<br>$F_{(1, 14)}$ =5.803<br><br><br>$F_{(1, 14)}$ =8.347<br>$F_{(1, 14)}$ =0.542	0.005**<br>0.030*<br><br><br>0.012*<br>0.474
J. SA training—activation of the VTA-DRN projections	Nose-pokes (three-way ANOVA):<br>Nose-poke(within)<br>Days (within)<br>Treatment (between)<br>Nose-poke * treatment<br>Days* treatment<br>Days * nose-poke<br>Days * nose-poke * treatment	 $F_{(1, 14)}$ =0.219<br>$F_{(9, 6)}$ =8.947<br>$F_{(1, 14)}$ =0.045<br>$F_{(1, 14)}$ =2.920<br>$F_{(9, 6)}$ =0.521<br>$F_{(9, 6)}$ =1.104<br>$F_{(9, 6)}$ =0.570	 0.647<br>0.007**<br>0.835<br>0.110<br>0.818<br>0.469<br>0.785
K. SA extinction—activation of the VTA-DRN projections	Nose-pokes (three-way ANOVA):<br>Nose-poke(within)<br>Days (within)<br>Treatment (between)<br>Nose-poke * treatment<br>Days* treatment<br>Nose-poke * days<br>Nose-poke * days* treatment	 $F_{(1, 14)}$ =43.838<br>$F_{(13, 2)}$ =23.001<br>$F_{(1, 14)}$ =0.042<br>$F_{(1, 14)}$ =0.194<br>$F_{(13, 2)}$ =2.421<br>$F_{(13, 2)}$ =1.723<br>$F_{(13, 2)}$ =2.070	 <0.001***<br>0.042*<br>0.840<br>0.666<br>0.330<br>0.427<br>0.372
L. Relapse test—activation of the VTA-DRN projections	Nose-pokes (two-way ANOVA):<br>Nose-poke(within)<br>Treatment (between)<br>Nose-poke * treatment	 $F_{(1, 14)}$ =120.372<br>$F_{(1, 14)}$ =1.542<br>$F_{(1, 14)}$ =1.163	 <0.001***<br>0.235<br>0.299

Supplementary Table 9. DRN-VTA^{DA} bidirectionally regulates heroin relapse

Figure number	Factor name	F-value	*p*-value
A. SA training—activation of DRN-VTA^{DA}	Nose-pokes (three-way ANOVA):		
	Nose-poke(within)	$F_{(1, 16}$=120.886	<0.001***
	Days (within)	$F_{(9, 8)}$ =3.529	0.045*
	Treatment (between)	$F_{(1, 16)}$ =0.070	0.795
	Nose-poke * treatment	$F_{(1, 16)}$ =0.431	0.521
	Days* treatment	$F_{(1, 16)}$ =0.338	0.937
	Days * nose-poke	$F_{(9, 8)}$ =28.341	<0.001***
	Days * nose-poke * treatment	$F_{(9, 8)}$ =3.126	0.062
B. SA extinction—activation of DRN-VTA^{DA}	Nose-pokes (three-way ANOVA):		
	Nose-poke(within)	$F_{(1, 16)}$ =48.753	<0.001***
	Days (within)	$F_{(6, 11)}$ =22.301	<0.001***
	Treatment (between)	$F_{(1, 16)}$ =1.784	0.200
	Nose-poke * treatment	$F_{(1, 16)}$ =0.019	0.892
	Days* treatment	$F_{(6, 11)}$ =1.387	0.302
	Nose-poke * days	$F_{(6, 11)}$ =7.905	0.002**
	Nose-poke * days* treatment	$F_{(6, 11)}$ =1.668	0.219
C. Relapse test—activation of DRN-VTA^{DA}	Nose-pokes (two-way ANOVA):		
	Nose-poke(within)	$F_{(1, 16)}$ =72.741	<0.001***
	Treatment (between)	$F_{(1, 16)}$ =20.221	<0.001***
	Nose-poke * treatment	$F_{(1, 16)}$ =16.892	<0.001***
	Simple effect analysis：		
	Active	$F_{(1, 16)}$ =27.993	<0.001***
	Inactiv	$F_{(1, 16)}$ =0.300	0.592
D. SA training—inhibition of DRN-VTA^{DA}	Nose-pokes (three-way ANOVA):		
	Nose-poke(within)	$F_{(1, 18)}$ =172.371	<0.001***
	Days (within)	$F_{(9, 10)}$ =5.859	0.005**
	Treatment (between)	$F_{(1, 18)}$ =0.224	0.642
	Nose-poke * treatment	$F_{(1, 18)}$ =0.481	0.497
	Days* treatment	$F_{(9, 10)}$ =1.210	0.383
	Days * nose-poke	$F_{(9, 10)}$ =17.396	<0.001***
	Days * nose-poke * treatment	$F_{(9, 10)}$ =0.687	0.708

E. SA extinction—inhibition of DRN-VTA^{DA}	Nose-pokes (three-way ANOVA):<br>Nose-poke(within)<br>Days (within)<br>Treatment (between)<br>Nose-poke * treatment<br>Days* treatment<br>Nose-poke * days<br>Nose-poke * days* treatment	 $F_{(1, 18)}$ =47.906<br>$F_{(6, 13)}$ =13.910<br>$F_{(1, 18)}$ =0.330<br>$F_{(1, 18)}$ =0.005<br>$F_{(6, 13)}$ =0.615<br>$F_{(6, 13)}$ =14.807<br>$F_{(6, 13)}$ =0.352	 <0.001***<br><0.001***<br>0.573<br>0.947<br>0.715<br><0.001***<br>0.896
F. Relapse test—inhibition of DRN-VTA^{DA}	Nose-pokes (two-way ANOVA):<br>Nose-poke(within)<br>Treatment (between)<br>Nose-poke * treatment<br><br>Simple effect analysis：<br>Active<br>Inactive	 $F_{(1, 18)}$ =52.008<br>$F_{(1, 18)}$ =10.423<br>$F_{(1, 18)}$ =5.682<br><br><br>$F_{(1, 18)}$ =11.151<br>$F_{(1, 18)}$ =0.338	 <0.001***<br>0.005**<br>0.028*<br><br><br>0.004**<br>0.568

Supplementary Table 10. Activation of the VP- or VP-VTA^{DA} pathway has no effect on cocaine relapse

Figure number	Factor name	F-value	*p*-value
A. SA training—activation of VP-VTA^{DA}	Nose-pokes (three-way ANOVA):		
	Nose-poke(within)	$F_{(1, 14)}$ =1.125	0.307
	Days (within)	$F_{(9, 6)}$ =7.978	0.010*
	Treatment (between)	$F_{(1, 14)}$ =0.024	0.879
	Nose-poke * treatment	$F_{(1, 14)}$ =0.589	0.456
	Days* treatment	$F_{(9, 6)}$ =0.227	0.977
	Days * nose-poke	$F_{(9, 6)}$ =3.268	0.082
	Days * nose-poke * treatment	$F_{(9, 6)}$ =0.466	0.854
B. SA extinction—activation of VP-VTA^{DA}	Nose-pokes (three-way ANOVA):		
	Nose-poke(within)	$F_{(1, 14)}$ =83.917	<0.001***
	Days (within)	$F_{(13, 2)}$ =115.353	0.009**
	Treatment (between)	$F_{(1, 14)}$ =0.011	0.918
	Nose-poke * treatment	$F_{(1, 14)}$ =0.002	0.968
	Days* treatment	$F_{(13, 2)}$ =0.885	0.647
	Nose-poke * days	$F_{(13, 2)}$ =7.193	0.129
	Nose-poke * days* treatment	$F_{(13, 2)}$ =1.456	0.480
C. Relapse test—activation of VP-VTA^{DA}	Nose-pokes (two-way ANOVA):		
	Nose-poke(within)	$F_{(1, 14)}$ =85.206	<0.001***
	Treatment (between)	$F_{(1, 14)}$ =0.116	0.739
	Nose-poke * treatment	$F_{(1, 14)}$ =0.123	0.731
D. SA training—activation of VS-VTA^{DA}	Nose-pokes (three-way ANOVA):		
	Nose-poke(within)	$F_{(1, 14)}$ =10.429	0.006**
	Days (within)	$F_{(9, 6)}$ =20.046	<0.001***
	Treatment (between)	$F_{(1, 14)}$ =0.006	0.937
	Nose-poke * treatment	$F_{(1, 14)}$ =0.610	0.448
	Days* treatment	$F_{(9, 6)}$ =0.307	0.945
	Days * nose-poke	$F_{(9, 6)}$ =1.672	0.274
	Days * nose-poke * treatment	$F_{(9, 6)}$ =0.435	0.874
E. SA extinction—activation of VS-VTA^{DA}	Nose-pokes (three-way ANOVA):		
	Nose-poke(within)	$F_{(1, 14)}$ =19.024	<0.001***
	Days (within)	$F_{(13, 2)}$ =30.861	0.032*

	Treatment (between)	$F_{(1, 14)}=0.130$	0.724
	Nose-poke * treatment	$F_{(1, 14)}=0.076$	0.787
	Days* treatment	$F_{(13, 2)}=0.425$	0.866
	Nose-poke * days	$F_{(13, 2)}=35.003$	0.028
	Nose-poke * days* treatment	$F_{(13, 2)}=5.872$	0.155
F. Relapse test—activation of VS-VTA^{DA}	Nose-pokes (two-way ANOVA):		
	Nose-poke(within)	$F_{(1, 14)}=100.429$	<0.001***
	Treatment (between)	$F_{(1, 14)}=0.099$	0.757
	Nose-poke * treatment	$F_{(1, 14)}=0.049$	0.828

Supplementary Table 11. Comparison of therapeutic effects between social reward and sucrose reward on cocaine seeking

Figure number	Factor name	F-value	*p*-value
A. SA training—Comparison of social reward and sucrose reward	Nose-pokes (three-way ANOVA):		
	Nose-poke(within)	$F_{(1, 25)}=156.963$	<0.001***
	Days (within)	$F_{(9, 17)}=2.319$	0.065
	Treatment (between)	$F_{(2, 25)}=0.167$	0.847
	Nose-poke * treatment	$F_{(2, 25)}=0.268$	0.456
	Days* treatment	$F_{(9, 18)}=0.721$	0.684
	Days * nose-poke	$F_{(9, 17)}=5.603$	0.001**
	Days * nose-poke * treatment	$F_{(9, 18)}=0.673$	0.723
B. SA extinction—Comparison of social reward and sucrose reward	Nose-pokes (three-way ANOVA):		
	Nose-poke(within)	$F_{(1, 25)}=149.251$	<0.001***
	Days (within)	$F_{(13, 13)}=16.368$	<0.001***
	Treatment (between)	$F_{(2, 25)}=0.044$	0.957
	Nose-poke * treatment	$F_{(2, 25)}=0.036$	0.965
	Days* treatment	$F_{(13, 14)}=1.517$	0.203
	Nose-poke * days	$F_{(13, 13)}=13.050$	<0.001***
	Nose-poke * days* treatment	$F_{(13, 14)}=1.925$	0.119
C. Relapse test—Comparison of social reward and sucrose reward	Nose-pokes (two-way ANOVA):		
	Nose-poke(within)	$F_{(1, 25)}=184.994$	<0.001***
	Treatment (between)	$F_{(2, 25)}=9.252$	0.001**
	Nose-poke * treatment	$F_{(2, 25)}=15.988$	<0.001***
	Simple effect analysis：		
	Active	$F_{(2, 25)}=13.344$	<0.001***
	Inactive	$F_{(2, 25)}=0.590$	0.562

Supplementary Table 12 Interacting with two juvenile partners is rewarding

Figure number	Factor name	F-value	*p*-value
A. Social SA training	Nose-pokes (three-way ANOVA):		
	Nose-poke(within)	$F_{(1, 14)}$ =1552.093	<0.001***
	Days (within)	$F_{(7, 8)}$ =1.951	0.185
	Treatment (between)	$F_{(1, 14)}$ =514.259	<0.001***
	Nose-poke * treatment	$F_{(1, 14)}$ =1165.105	<0.001***
	Days* treatment	$F_{(7, 8)}$ =3.203	0.063
	Days * nose-poke	$F_{(7, 8)}$ =3.930	0.037*
	Days * nose-poke * treatment	$F_{(7, 8)}$ =3.781	0.041*
	Simple Simple Effects Analysis		
	Active:		
	Day1	$F_{(1, 14)}$ =31.909	<0.001***
	Day2	$F_{(1, 14)}$ =45.805	<0.001***
	Day3	$F_{(1, 14)}$ =188.597	<0.001***
	Day4	$F_{(1, 14)}$ =283.703	<0.001***
	Day5	$F_{(1, 14)}$ =605.015	<0.001***
	Day6	$F_{(1, 14)}$ =278.633	<0.001***
	Day7	$F_{(1, 14)}$ =354.792	<0.001***
	Day8	$F_{(1, 14)}$ =374.163	<0.001***
	Inactive:		
	Day1	$F_{(1, 14)}$ =0.055	0.818
	Day2	$F_{(1, 14)}$ =1.524	0.237
	Day3	$F_{(1, 14)}$ =0.163	0.692
	Day4	$F_{(1, 14)}$ =0.586	0.457
	Day5	$F_{(1, 14)}$ =4.817	0.056
	Day6	$F_{(1, 14)}$ =0.116	0.739
	Day7	$F_{(1, 14)}$ =0.193	0.667
	Day8	$F_{(1, 14)}$ =0.000	1.000
B. Social-engram ICSS (Day1-4)	three-way ANOVA:		
	Laser (within)	$F_{(1, 14)}$ =109.015	<0.001***
	Days (within)	$F_{(3, 12)}$ =3.375	0.055

	Treatment (between)	$F_{(1, 14)}$ =117.612	<0.001***
	Laser * treatment	$F_{(1, 14)}$ =99.027	<0.001***
	Days* treatment	$F_{(3, 12)}$ =5.094	0.017*
	Days * Laser	$F_{(3, 12)}$ =4.193	0.030*
	Days * Laser * treatment	$F_{(3, 12)}$ =5.764	0.011*
	Simple Simple Effects Analysis		
	Active:		
	Day1	$F_{(1, 14)}$ =51.553	<0.001***
	Day2	$F_{(1, 14)}$ =72.520	<0.001***
	Day3	$F_{(1, 14)}$ =55.768	<0.001***
	Day4	$F_{(1, 14)}$ =62.311	<0.001***
	Inactive:		
	Day1	$F_{(1, 14)}$ =5.737	0.061
	Day2	$F_{(1, 14)}$ =7.353	0.057
	Day3	$F_{(1, 14)}$ =8.385	0.052
	Day4	$F_{(1, 14)}$ =7.304	0.057
C. Social-engram ICSS (Day5-8)	three-way ANOVA:		
	Laser (within)	$F_{(1, 14)}$ =66.491	<0.001***
	Days (within)	$F_{(3, 12)}$ =2.807	0.085
	Treatment (between)	$F_{(1, 14)}$ =113.108	<0.001***
	Laser * treatment	$F_{(1, 14)}$ =64.717	<0.001***
	Days* treatment	$F_{(3, 12)}$ =6.941	0.006**
	Days * Laser	$F_{(3, 12)}$ =33.625	<0.001***
	Days * Laser * treatment	$F_{(3, 12)}$ =21.248	<0.001***
	Simple Simple Effects Analysis		
	Active:		
	Day5	$F_{(1, 14)}$ =45.567	<0.001***
	Day6	$F_{(1, 14)}$ =68.480	<0.001***
	Day7	$F_{(1, 14)}$ =33.370	<0.001***
	Day8	$F_{(1, 14)}$ =71.161	<0.001***
	Inactive:		

	Day5<br>Day6<br>Day7<br>Day8	$F_{(1, 14)}$ =26.714<br>$F_{(1, 14)}$ =22.962<br>$F_{(1, 14)}$ =10.610<br>$F_{(1, 14)}$ =23.867	<0.001***<br><0.001***<br><0.001***<br><0.001***
D. Social-engram ICSS (Average)	Two-way ANOVA:<br>Laser (within)<br>Treatment (between)<br>Laser * treatment<br><br>Simple Effects Analysis<br>ChR2:<br>mCherry:	 $F_{(1, 14)}$ =107.809<br>$F_{(1, 14)}$ =132.657<br>$F_{(1, 14)}$ =103.654<br><br><br>$F_{(1, 14)}$ =211.442<br>$F_{(1, 14)}$ =0.020	 <0.001***<br><0.001***<br><0.001***<br><br><br><0.001***<br>0.888

Supplementary Table 13 The protective effect of acute social interaction against drug seeking is transient

Figure number	Factor name	F-value	*p*-value
A. SA training—effect is transient	Nose-pokes (three-way ANOVA):		
	Nose-poke(within)	$F_{(1, 15)}$ =24.548	<0.001***
	Days (within)	$F_{(9, 7)}$ =23.077	<0.001***
	Treatment (between)	$F_{(1, 15)}$ =0.082	0.778
	Nose-poke * treatment	$F_{(1, 15)}$ =0.016	0.900
	Days* treatment	$F_{(9, 7)}$ =0.261	0.967
	Days * nose-poke	$F_{(9, 7)}$ =0.897	0.571
	Days * nose-poke * treatment	$F_{(9, 7)}$ =0.429	0.882
B. SA extinction—effect is transient	Nose-pokes (three-way ANOVA):		
	Nose-poke(within)	$F_{(1, 15)}$ =18.300	0.001**
	Days (within)	$F_{(13, 3)}$ =29.352	0.009**
	Treatment (between)	$F_{(1, 15)}$ =0.029	0.866
	Nose-poke * treatment	$F_{(1, 15)}$ =0.003	0.961
	Days* treatment	$F_{(13, 3)}$ =0.191	0.986
	Nose-poke * days	$F_{(13, 3)}$ =4.313	0.127
	Nose-poke * days* treatment	$F_{(13, 3)}$ =0.992	0.579
C. Relapse test—effect is transient	Nose-pokes (three-way ANOVA):		
	Nose-poke(within)	$F_{(1, 15)}$ =265.970	<0.001***
	Days (within)	$F_{(3,1 3)}$ =54.455	<0.001***
	Treatment (between)	$F_{(1, 15)}$ =19.736	<0.001***
	Nose-poke * treatment	$F_{(1, 15)}$ =11.230	0.004**
	Days* treatment	$F_{(3,1 3)}$ =60.489	<0.001***
	Nose-poke * days	$F_{(3,1 3)}$ =25.057	<0.001***
	Nose-poke * days* treatment	$F_{(3, 13)}$ =36.033	<0.001***
	Simple Simple Effects Analysis		
	Active:		
	Day53	$F_{(1, 15)}$ =0.537	0.475
	Day54	$F_{(1, 15)}$ =62.634	<0.001***
	Day55	$F_{(1, 15)}$ =2.441	0.139
	Day56	$F_{(1, 15)}$ =9.819	0.007**
	Inactive:		

	Day53<br>Day54<br>Day55<br>Day56	$F_{(1, 15)}$ =2.194<br>$F_{(1, 15)}$ =3.129<br>$F_{(1, 15)}$ =3.967<br>$F_{(1, 15)}$ =0.157	0.159<br>0.097<br>0.065<br>0.697

Supplementary Table 14 Pair-housed animals exhibited accelerated extinction and reduced cocaine seeking

Figure number	Factor name	F-value	*p*-value
A. SA training—Pair-housed	Nose-pokes (three-way ANOVA):		
	Nose-poke(within)	$F_{(1, 17)}$ =215.645	<0.001***
	Days (within)	$F_{(9, 9)}$ =5.236	0.011*
	Treatment (between)	$F_{(1, 17)}$ =0.188	0.670
	Nose-poke * treatment	$F_{(1, 17)}$ =0.005	0.942
	Days* treatment	$F_{(9, 9)}$ =0.579	0.786
	Days * nose-poke	$F_{(9, 9)}$ =4.468	0.018
	Days * nose-poke * treatment	$F_{(9, 9)}$ =0.305	0.954
B. SA extinction—Pair-housed	Nose-pokes (three-way ANOVA):		
	Nose-poke(within)	$F_{(1, 17)}$ =174.355	<0.001***
	Days (within)	$F_{(13, 5)}$ =43.565	<0.001***
	Treatment (between)	$F_{(1, 17)}$ =12.849	0.002**
	Nose-poke * treatment	$F_{(1, 17)}$ =0.467	0.504
	Days* treatment	$F_{(13, 5)}$ =14.720	0.004**
	Nose-poke * days	$F_{(13, 5)}$ =9.157	0.012*
	Nose-poke * days* treatment	$F_{(13, 5)}$ =4.799	0.047*
	Simple Simple Effects Analysis		
	Active:		
	Day12	$F_{(1, 17)}$ =3.231	0.090
	Day13	$F_{(1, 17)}$ =5.929	0.026*
	Day14	$F_{(1, 17)}$ =10.783	0.004**
	Day15	$F_{(1, 17)}$ =17.660	0.001**
	Day16	$F_{(1, 17)}$ =22.291	<0.001***
	Day17	$F_{(1, 17)}$ =35.234	<0.001***
	Day18	$F_{(1, 17)}$ =29.718	<0.001***
	Day19	$F_{(1, 17)}$ =22.356	<0.001***
	Day20	$F_{(1, 17)}$ =21.323	<0.001***
	Day21	$F_{(1, 17)}$ =16.930	0.001**
	Day22	$F_{(1, 17)}$ =15.394	0.001**
	Day23	$F_{(1, 17)}$ =0.686	0.419
	Day24	$F_{(1, 17)}$ =0.045	0.830

	Day25	$F_{(1, 17)}$ =0.048	0.830
	Inactive:		
	Day12	$F_{(1, 17)}$ =0.049	0.827
	Day13	$F_{(1, 17)}$ =0.127	0.726
	Day14	$F_{(1, 17)}$ =0.085	0.775
	Day15	$F_{(1, 17)}$ =0.026	0.875
	Day16	$F_{(1, 17)}$ =0.001	0.972
	Day17	$F_{(1, 17)}$ =0.000	0.988
	Day18	$F_{(1, 17)}$ =0.312	0.584
	Day19	$F_{(1, 17)}$ =0.832	0.374
	Day20	$F_{(1, 17)}$ =0.686	0.419
	Day21	$F_{(1, 17)}$ =0.820	0.378
	Day22	$F_{(1, 17)}$ =0.045	0.834
	Day23	$F_{(1, 17)}$ =0.040	0.843
	Day24	$F_{(1, 17)}$ =0.048	0.830
	Day25	$F_{(1, 17)}$ =0.001	0.979
C. Relapse test—Pair-housed	Nose-pokes (two-way ANOVA):		
	Nose-poke(within)	$F_{(1, 17)}$ =118.475	<0.001***
	Treatment (between)	$F_{(1, 17)}$ =18.874	<0.001***
	Nose-poke * treatment	$F_{(1, 17)}$ =14.861	0.001**
	Simple Effects Analysis		
	Active:	$F_{(1, 17)}$ =17.227	0.001**
	Inactive:	$F_{(1, 17)}$ =0.048	0.828

Supplementary Table 15 Activating Drug-seeking-Ens^{DA} suppress feeding behaviors

Figure number	Factor name	F-value	*p*-value
A. Relapse test—Activating Social-reward-Ens^{DA}	Refeeding (Two-way ANOVA):<br>Times (within)<br>Treatment (between)<br>Nose-poke * treatment	 $F_{(2,1 3)}$ =217.558<br>$F_{(2, 13)}$ =17.109<br>$F_{(2, 13)}$ =1.914	 <0.001***<br>0.001<br>0.187
B. Relapse test—Activating Drug-seeking-Ens^{DA}	Refeeding (Two-way ANOVA):<br>Times (within)<br>Treatment (between)<br>Nose-poke * treatment<br><br>Simple Effects Analysis:<br>1h<br>2h<br>4h	 $F_{(2,1 3)}$ =158.349<br>$F_{(2, 13)}$ =1.748<br>$F_{(2, 13)}$ =5.545<br><br><br>$F_{(1, 14)}$ =33.800<br>$F_{(1, 14)}$ =10.874<br>$F_{(1, 14)}$ =1.994	 <0.001***<br>0.207<br>0.018*<br><br><br><0.001***<br>0.005**<br>0.180
