# Supplementary material for: Social reward outcompetes drug seeking dopaminergic ensembles to prevent relapse
Source: Nat Commun. 2026 Apr 3;17:3462. doi: 10.1038/s41467-026-71357-4 (PMC13076667; doi:10.1038/s41467-026-71357-4)
Supplement: Supplementary file 2 — Reporting Summary [file 41467_2026_71357_MOESM2_ESM.pdf]

nature portfolio

Corresponding author(s): Yan-Xue Xue

Last updated by author(s): Jan 20, 2026

Reporting Summary

Nature Portfolio wishes to improve the reproducibility of the work that we publish. This form provides structure for consistency and transparency in reporting. For further information on Nature Portfolio policies, see our Editorial Policies and the Editorial Policy Checklist.

Please do not complete any field with "not applicable" or n/a. Refer to the help text for what text to use if an item is not relevant to your study.
For final submission: please carefully check your responses for accuracy; you will not be able to make changes later.

Statistics

For all statistical analyses, confirm that the following items are present in the figure legend, table legend, main text, or Methods section.

n/a	Confirmed	
☐	☒	The exact sample size (*n*) for each experimental group/condition, given as a discrete number and unit of measurement
☐	☒	A statement on whether measurements were taken from distinct samples or whether the same sample was measured repeatedly
☐	☒	The statistical test(s) used AND whether they are one- or two-sided<br>*Only common tests should be described solely by name; describe more complex techniques in the Methods section.*
☒	☐	A description of all covariates tested
☐	☒	A description of any assumptions or corrections, such as tests of normality and adjustment for multiple comparisons
☐	☒	A full description of the statistical parameters including central tendency (e.g. means) or other basic estimates (e.g. regression coefficient) AND variation (e.g. standard deviation) or associated estimates of uncertainty (e.g. confidence intervals)
☐	☒	For null hypothesis testing, the test statistic (e.g. *F*, *t*, *r*) with confidence intervals, effect sizes, degrees of freedom and *P* value noted<br>*Give P values as exact values whenever suitable.*
☒	☐	For Bayesian analysis, information on the choice of priors and Markov chain Monte Carlo settings
☐	☒	For hierarchical and complex designs, identification of the appropriate level for tests and full reporting of outcomes
☐	☒	Estimates of effect sizes (e.g. Cohen's *d*, Pearson's *r*), indicating how they were calculated

Our web collection on statistics for biologists contains articles on many of the points above.

Software and code

Policy information about availability of computer code

Data collection	Intravenous cocaine and heroin self-administration data: Commercialized Software from AniLab;<br>Fiber photometry data: Commercialized Software from Inper;<br>Single-neuron calcium imaging: Commercialized Software from Thinkerbiotech;<br>Whole-cell recording data: Commercialized Software Clampex10.5;<br>Histological image data: Commercialized software OlyVIA.
Data analysis	MATLAB 2022, SPSS 2022; ImageJ; and Prism 9.1.0

For manuscripts utilizing custom algorithms or software that are central to the research but not yet described in published literature, software must be made available to editors and reviewers. We strongly encourage code deposition in a community repository (e.g. GitHub). See the Nature Portfolio guidelines for submitting code & software for further information.

Data

Policy information about availability of data

All manuscripts must include a data availability statement. This statement should provide the following information, where applicable:

- Accession codes, unique identifiers, or web links for publicly available datasets
- A description of any restrictions on data availability
- For clinical datasets or third party data, please ensure that the statement adheres to our policy

The raw data used in this paper are available from the corresponding author upon request.

Research involving human participants, their data, or biological material

Policy information about studies with human participants or human data. See also policy information about sex, gender (identity/presentation), and sexual orientation and race, ethnicity and racism.

Reporting on sex and gender	This information has not been collected
Reporting on race, ethnicity, or other socially relevant groupings	This information has not been collected
Population characteristics	This information has not been collected
Recruitment	This information has not been collected
Ethics oversight	This information has not been collected

Note that full information on the approval of the study protocol must also be provided in the manuscript.

Field-specific reporting

Please select the one below that is the best fit for your research. If you are not sure, read the appropriate sections before making your selection.

☒ Life sciences ☐ Behavioural & social sciences ☐ Ecological, evolutionary & environmental sciences

For a reference copy of the document with all sections, see nature.com/documents/nr-reporting-summary-flat.pdf

Life sciences study design

All studies must disclose on these points even when the disclosure is negative.

Sample size	Behavioral data for each condition were sampled from 8 to 10 rats. For fiber photometry recordings, the sample sizes were from 5 to 10 rats. Single-neuron calcium imaging data were sampled from 3 to 6 rats. Immunocytochemistry data for each condition were sampled from tissue sections of 4 to 5 rats. These sample sizes align with those reported in previous studies (PMID: 22499948, PMID: 26169171, PMID: 28648649, PMID: 37657441, PMID: 36563158).
Data exclusions	In the experiments involving intravenous catheter implantation, such as cocaine and heroin self-administration experiments, 16 rats were excluded due to death from infection or blood loss, and 43 rats were excluded due to issues with the catheter, such as leakage or obstruction. For the fiber photometry and single-neuron calcium imaging manipulation experiments, 18 rats were excluded due to incorrect fiber or lens placement. For chemogenetic manipulation, 17 rats were excluded due to histological evaluation of the virus infusion target.
Replication	Experiments were performed with sufficient rats per group to demonstrate statistical significance.
Randomization	Rats were randomly assigned into control or different experimental groups.
Blinding	We were blinded for the fiber photometry recordings, single-neuron calcium imaging, and some electrophysiological recording but not for the behavioral experiments.

Reporting for specific materials, systems and methods

We require information from authors about some types of materials, experimental systems and methods used in many studies. Here, indicate whether each material, system or method listed is relevant to your study. If you are not sure if a list item applies to your research, read the appropriate section before selecting a response.

Materials & experimental systems

n/a	Involved in the study
☐	☒ Antibodies
☒	☐ Eukaryotic cell lines
☒	☐ Palaeontology and archaeology
☐	☒ Animals and other organisms
☒	☐ Clinical data
☒	☐ Dual use research of concern
☒	☐ Plants

Methods

n/a	Involved in the study
☒	☐ ChIP-seq
☒	☐ Flow cytometry
☒	☐ MRI-based neuroimaging

Antibodies

Antibodies used

Rabbit anti-tyrosine hydroxylase (Millipore, Cat# AB152; RRID:AB_390204),
Rabbit anti-c-Fos (Abcam, Cat# ab190289; RRID:AB_2737414),
Mouse anti-c-Fos (Abcam, Cat# ab208942; RRID:AB_2747772),
Goat anti-Rabbit IgG (H+L) Highly Cross-Adsorbed Secondary Antibody, Alexa Fluor 488 (Invitrogen, Cat# A-11034; RRID:AB_2576217),
Goat anti-Rabbit IgG (H+L) Highly Cross-Adsorbed Secondary Antibody, Alexa Fluor 546 (Invitrogen, Cat# A-11035; RRID:AB_2534093),
Goat anti-Rabbit IgG (H+L) Highly Cross-Adsorbed Secondary Antibody, Alexa Fluor™ 647 (Invitrogen, Cat# A-21245; RRID:AB_2535813),
Donkey anti-Mouse IgG (H+L) Highly Cross-Adsorbed Secondary Antibody, Alexa Fluor 647 (Invitrogen, Cat# A-31571; RRID:AB_162542),

Validation

(1) Rabbit anti-tyrosine hydroxylase (Millipore, Cat# AB152; RRID:AB_390204)
Description: This antibody detects the level of tyrosine hydroxylase (TH) and has been published and validated for use in ELISA, IF, IH, IH(P), IP, and WB. It reacts with most mammalian and many non-mammalian species, including human, feline, ferret, rat, squid, mouse, and more. It is a useful marker for dopaminergic and noradrenergic neurons.
Species Reactivity: Human, feline, ferret, rat, squid, mouse, and others.
Reference: PMID: 29398114.
(2) Rabbit anti-c-Fos (Abcam, Cat# ab190289; RRID:AB_2737414)
Description: This antibody detects the level of c-Fos and has been published and validated for use in various experimental techniques, including ELISA, IF, IH, IH(P), IP, and WB. It reacts with most mammalian and many non-mammalian species, including human, feline, ferret, rat, squid, mouse, and more. It exhibits reactivity with most mammalian species and many non-mammalian species, such as human, feline, ferret, rat, and mouse, among others. c-Fos is a well-characterized immediate early gene product, often used as a marker for neuronal activation and cellular responses to various stimuli.
Species Reactivity: Human, rat, mouse, and others.
Reference: PMID: 36753548.
(3) Mouse anti-c-Fos (Abcam, Cat# ab208942; RRID:AB_2747772)
Description: This antibody detects the level of c-Fos and has been published and validated for use in various experimental techniques, including ELISA, IF, IH, IH(P), IP, and WB. It reacts with most mammalian and many non-mammalian species, including human, feline, ferret, rat, squid, mouse, and more. It exhibits reactivity with most mammalian species and many non-mammalian species, such as human, feline, ferret, rat, and mouse, among others. c-Fos is a well-characterized immediate early gene product, often used as a marker for neuronal activation and cellular responses to various stimuli.
Species Reactivity: Human, mouse, rat, and others.
Reference: PMID: 37248289.
(4) Goat anti-Rabbit IgG (H+L) Highly Cross-Adsorbed Secondary Antibody, Alexa Fluor 488 (Invitrogen, Cat# A-11034; RRID:AB_2576217)
Description: This secondary antibody is highly cross-adsorbed and conjugated with Alexa Fluor 488, a bright green-fluorescent dye. It is designed for use in immunofluorescence, flow cytometry, and other applications where green fluorescence detection is required.
Species Reactivity: Rabbit IgG.
Reference: PMID: 40489346.
(5) Goat anti-Rabbit IgG (H+L) Highly Cross-Adsorbed Secondary Antibody, Alexa Fluor 546 (Invitrogen, Cat# A-11035; RRID:AB_2534093)
Description: This secondary antibody is highly cross-adsorbed and conjugated with Alexa Fluor 546, a bright orange-fluorescent dye. It is designed for use in immunofluorescence, flow cytometry, and other applications where green fluorescence detection is required.
Species Reactivity: Rabbit IgG.
Reference: PMID: 40759652.
(6) Goat anti-Rabbit IgG (H+L) Highly Cross-Adsorbed Secondary Antibody, Alexa Fluor™ 647 (Invitrogen, Cat# A-21245; RRID:AB_2535813)
Description: This secondary antibody is highly cross-adsorbed and conjugated with Alexa Fluor 647, a near-infrared-fluorescent dye. It is designed for use in immunofluorescence, flow cytometry, and other applications where green fluorescence detection is required.
Species Reactivity: Rabbit IgG.
Reference: PMID: 40769979.
(7) Donkey anti-Mouse IgG (H+L) Highly Cross-Adsorbed Secondary Antibody, Alexa Fluor 647 (Invitrogen, Cat# A-31571; RRID:AB_162542)
Description: This secondary antibody is highly cross-adsorbed and conjugated with Alexa Fluor 647, a near-infrared-fluorescent dye. It is designed for use in immunofluorescence, flow cytometry, and other applications where green fluorescence detection is required.
Species Reactivity: Mouse IgG.

Reference: PMID: 40727930.

Animals and other research organisms

Policy information about studies involving animals; ARRIVE guidelines recommended for reporting animal research, and Sex and Gender in Research

Laboratory animals	Male Sprague-Dawley rats (280–300g; Beijing Vital River, China) were group-housed (5/cage) for one week, then singly housed post-surgery. Rats were kept on a 12h light/dark cycle (lights on at 20:00) with ad libitum food and water. Behavioral tests were conducted during the dark phase.
Wild animals	No wild animals were used in this study.
Reporting on sex	Only male rats were used in the present study, and sex was not included as a variable in the study design or statistical analysis.
Field-collected samples	No field-collected samples were used in this research
Ethics oversight	All procedures followed the guidelines set forth by the Regulation for the Administration of Affairs Concerning Experimental Animals (China, 1988) and the National Institutes of Health Guide for the Care and Use of Laboratory Animals, with approval by the Biomedical Ethics Committee for Animal Use and Protection of Peking University.

Note that full information on the approval of the study protocol must also be provided in the manuscript.

This checklist template is licensed under a Creative Commons Attribution 4.0 International License, which permits use, sharing, adaptation, distribution and reproduction in any medium or format, as long as you give appropriate credit to the original author(s) and the source, provide a link to the Creative Commons license, and indicate if changes were made. The images or other third party material in this article are included in the article's Creative Commons license, unless indicated otherwise in a credit line to the material. If material is not included in the article's Creative Commons license and your intended use is not permitted by statutory regulation or exceeds the permitted use, you will need to obtain permission directly from the copyright holder. To view a copy of this license, visit http://creativecommons.org/licenses/by/4.0/
